# Genome-Wide Functional Divergence after the Symbiosis of Proteobacteria with Insects Unraveled through a Novel Computational Approach

**DOI:** 10.1371/journal.pcbi.1000344

**Published:** 2009-04-03

**Authors:** Christina Toft, Tom A. Williams, Mario A. Fares

**Affiliations:** Department of Genetics, Trinity College Dublin, University of Dublin, Dublin, Ireland; University of Oxford, United Kingdom

## Abstract

Symbiosis has been among the most important evolutionary steps to generate biological complexity. The establishment of symbiosis required an intimate metabolic link between biological systems with different complexity levels. The strict endo-cellular symbiotic bacteria of insects are beautiful examples of the metabolic coupling between organisms belonging to different kingdoms, a eukaryote and a prokaryote. The host (eukaryote) provides the endosymbiont (prokaryote) with a stable cellular environment while the endosymbiont supplements the host's diet with essential metabolites. For such communication to take place, endosymbionts' genomes have suffered dramatic modifications and reconfigurations of proteins' functions. Two of the main modifications, loss of genes redundant for endosymbiotic bacteria or the host and bacterial genome streamlining, have been extensively studied. However, no studies have accounted for possible functional shifts in the endosymbiotic proteomes. Here, we develop a simple method to screen genomes for evidence of functional divergence between two species clusters, and we apply it to identify functional shifts in the endosymbiotic proteomes. Despite the strong effects of genetic drift in the endosymbiotic systems, we unexpectedly identified genes to be under stronger selective constraints in endosymbionts of aphids and ants than in their free-living bacterial relatives. These genes are directly involved in supplementing the host's diet with essential metabolites. A test of functional divergence supports a strong relationship between the endosymbiosis and the functional shifts of proteins involved in the metabolic communication with the insect host. The correlation between functional divergence in the endosymbiotic bacterium and the ecological requirements of the host uncovers their intimate biochemical and metabolic communication and provides insights on the role of symbiosis in generating species diversity.

## Author Summary

Biological complexity has emerged on earth by the combination of living forms. This combination, called symbiosis, had to overcome the problems caused by the uncoupled metabolisms of the organisms involved. One way to do so was through the loss of genes that were no longer needed for the endosymbiont in the protected cellular environment provided by the host. Another step necessary to adjust both metabolisms was through the change in the function of bacterial proteins to perform new roles in the symbiotic system. In this article, we test such events in symbiotic systems involving an insect and a bacterium by developing a new and simple method to identify proteome-wide functional shifts. Our results show that most of the functional changes occurred at genes involved in metabolic communication with the host and are correlated with the host's ecological traits.

## Introduction

One of the most fascinating puzzles in evolutionary biology is how variability at the gene and protein level leads to the generation of new species. Inextricably linked to this question are two main issues that remain the focus of heated debates and arduous investigation: i) The extent of variation in a protein's function; and ii) the relationship between the variability of a protein's sequence and its function. Organism lineages generally evolve under strict negative selection (purifying selection), with bursts of adaptive mutations becoming punctually fixed in the population. Negative selection generally removes functionally/structurally destabilizing mutations, resulting in protein functional stasis [1]. Conversely, diversifying selection contributes to the emergence of new protein functions [2]. Protein structure is the major determinant of function and it is expected that the structural stability of a protein should provide functional stability. In fact, recent evidence suggests that structural robustness to mistranslation errors is the factor determining protein's evolutionary rate [3]. Consequently, mutations will only be fixed at amino acid sites with no structural importance, while selection will remove mutations destabilizing protein's structure [4],[5]. Selection shifts at particular sites that may affect protein structure and function can lead to functional divergence [6]. The evolutionary constraints whereby selection shifts occur range between neutrality [7] and selection due to functional divergence [8],[9].

There are several scenarios under which change in selective pressures may occur, with gene duplication being the most prominent case [10]–[18]. Revolutionary changes in the organism's lifestyle may also lead to proteome functional divergence and to the consequent emergence of new species. Symbiosis of proteobacteria with insects is a striking example of revolutionized change in the lifestyle of an organism (a free-living bacterium became an endocellular symbiotic bacterium) that has been directly linked to the generation of species diversity. Interspecific interactions act in concert with environmental changes to maintain or generate species diversification [19],[20]. Examples of the role of interspecific interactions is the case of adaptive radiation of plant-pollinator mutualisms in which plant traits related to related to reproductive isolation become subject to natural selection [19],[21],[22]. In geological terms, adaptive radiation has been repeatedly accelerated by the symbiosis of two different organisms [23]. In extreme symbiosis cases, such as the endosymbiotic bacteria of insects, symbiosis involved a dramatic lifestyle change that was accompanied by dramatic population-genetics events including strong bottlenecks to the bacterial effective population sizes during intergenerational transmission. In some biological systems, such as in the symbiosis between aphid insects and the bacterium *Buchnera aphidicola sp.*, the effect of bottlenecking can be dramatic due to the clonal and vertical transmission of the bacteria. This bottlenecking results in strong genetic drift and the accelerated fixation of mutations, leading to profound genomic and metabolic remodeling in endosymbiotic bacteria. For example, the switch of bacteria from a free lifestyle to symbiosis with a qualitatively more complex organism may lead to dramatic changes in genomic and metabolic architecture. Indeed, intracellular life may render most of the biological processes related to extra-cellular survival redundant. This gene redundancy may have similar evolutionary consequences to gene duplication in that selection relaxes over such genes allowing the fixation of new mutations despite their slightly destabilizing effects or their slightly advantageous consequences. Furthermore, the proteome/interactome and metabolism of the bacterium may change during the establishment of the metabolic interlink between host and bacterium [24]. In particular, the stable environment provided by the insect aphid to the endosymbiotic bacterium *Buchnera* and the presence in some cases of secondary endosymbionts collaborating in close metabolic intimacy with the host renders most of the genes in the endosymbiont redundant [25],[26]. The consequent relaxed constraints on these genes, in addition to the strong intergenerational bottlenecks these bacteria undergo [27], has resulted in a well-characterized syndrome for endosymbiosis. Among the genomic characteristics of endosymbiotic bacteria are an AT enrichment and accelerated protein evolutionary rates [27]–[33], genome reduction (for example see [34],[35]), low levels of intra-specific polymorphism [33],[36], and decreased stability of RNAs [37] and of proteins [38]. Beside of all these effects, we also expect ample opportunity for functional divergence in these bacteria, since: i) strong genetic drift allows the neutral fixation of mildly deleterious mutations that may become functionally interesting when ameliorated by compensatory mutations; and ii) symbiosis may have favored the emergence of new functions enabling biochemical communication with the host as well as saving metabolic energy.

An example of genomic economization is the flagella assembly pathway in bacteria that is also responsible for protein export in free-living bacteria. It has been shown that endosymbiotic bacteria of insects such as *Buchnera* are non-motile and yet they express many of the hook and basal body genes of the flagella [39], supporting previous suggestions of the specialization of these genes in export of proteins from the host to the bacterium [40]. Recently, we have conducted an exhaustive evolutionary analysis of the flagella genes in endosymbiotic bacteria of insects and showed that indeed some genes may have changed their function towards protein export [26]. Identification of functional divergence is key in understanding the metabolic communication between the host and the endosymbiont. However, the detection of adaptive evolution caused by functional divergence is usually hampered by the fact that genetic drift in these bacteria may produce similar evolutionary patterns. Therefore, standard statistical methods cannot disentangle functional divergence from genetic drift effects and alternative strategies are needed.

To better understand the scenarios under which the endosymbiotic bacteria of insects adapted to a dramatically different lifestyle in comparison with their closest free-living relatives, we here conduct a genome-wide analysis of functional divergence in the endosymbiont of aphids and endosymbionts of carpenter ants using a novel and simple statistical approach.

## Results

To investigate the relationship between endosymbiosis and the shift in the nucleotide substitution rates we first estimated synonymous (*d_S_*) and non-synonymous (*d_N_*) pairwise substitutions as well as the ratio between both these estimates (*ω* = *d_N_*/*d_S_*) in endosymbiotic bacteria and in their free-living relatives. To generalize our conclusions, we present results from the two endosymbiotic systems, *Buchnera* and *Blochmannia sp.* (hereon we will use the genus name to refer to these endosymbionts *Buchnera* and *Blochmannia*), in each one of the sub-sections. To compare endosymbiotic evolutionary rates we used the comparisons *BAp*-*BSg* and *Bf*-*Bp* to their free-living relatives *Ec*-*St* because these divergence events occurred at equivalent times. Therefore, the comparisons are appropriate despite possible pressures on synonymous sites.

### Differential Selective Constraints in Endosymbiotic Genomes

*Buchnera sp.* genomes experienced relaxed constraints after the establishment of endosymbiosis with aphids because the estimated number of substitutions increased proportionally in synonymous and non-synonymous sites (Table 1). For example, *d_N_* in endosymbionts (*d_Ne_*) increased on average fivefold when compared to *d_N_* in free-living bacteria (*d_Nf_*) (Median ratio *R*(*d_N_*) = *d_Ne_*/*d_Nf_* = 5.118). Likewise, *d_Se_* increased on average three fold when compared to *d_Sf_* (*R*(*d_S_*) = 3.329). On average, then, both types of sites experienced relaxed constraints after the symbiosis of bacteria with aphids, but this relaxation was more significant at non-synonymous sites, further highlighting the importance of genetic drift during the evolution of endosymbiotic bacteria. The endosymbiont of carpenter ants presented similar relaxed constraints at synonymous sites but much more relaxed constraints at non-synonymous sites when compared to *Buchnera sp.* (Table 1).

**Table 1:** Increments of selective constraints in endosymbiotic bacteria of insects.

Data	Median			
	*Buchnera sp.*		*Blochmannia sp.*	
	R(*d_N_*)^a^	R(*d_S_*)^b^	R(*d_N_*)	R(*d_S_*)
Full Dataset	5.118±2.097	3.329±2.018	7.458±4.511	3.568±2.395
R(*ω*)^c^<1	3.288±1.506	5.344±3.470	4.393±2.531	6.738±3.554
R(*ω*)≥1	5.302±3.639	2.766±0.211	8.1353±5.316	3.146±2.064

a Ratio between the rate of non-synonymous substitutions per site in endosymbiotic bacteria and that of their free-living bacteria.

b Ratio between the rate of synonymous substitutions per site in endosymbiotic bacteria and that of their free-living bacteria.

c Ratio between the non-synonymous-to-synonymous rates ratio of endosymbiotic bacteria and that of their free-living relatives.

**Figure 1:**
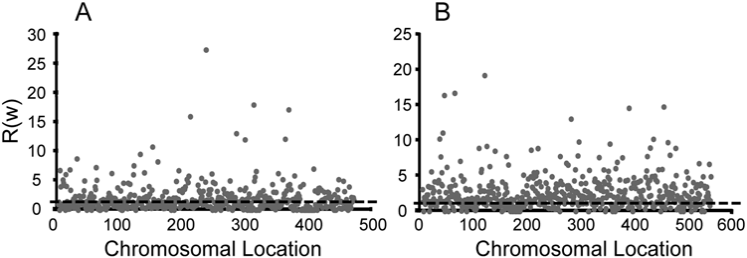
Constraints operating in endosymbiotic bacteria of aphids (A) and carpenter ants (B) in comparison with their free-living relative bacteria *Escherichia coli* and *Salmonella typhimurium*. We compared the constraints operating in protein-coding genes between endosymbiotic and free-living bacteria by dividing the non-synonymous-to-synonymous rates ratio of endosymbionts (*ω_e_*) by that of their free-living relatives (*ω_f_*) and we called this ratio R(*ω*) [R(*ω*) = *ω_e_*/*ω_f_*; represented in the Y-axis). We plotted genes according to their position in the bacterial chromosome (X-axis). We also indicate R(*ω*) = 1 since this is the value at which genes have not changed their selective constraints.

Contrary to the expectation of a genome-wide relaxation of constraints after symbiosis, we found that some genes showed increased selection pressures in the endosymbiotic lineages, with greater selection intensities in endosymbionts (*ω_e_*) than in their free-living relatives (*ω_f_*) [*R*(*ω*) = ω_e_/ω_f_<1]. The number of genes showing such ratios was significant with as much as 29.67% of the genes (151 out of 509 genes) and 16.98% of genes (91 out of the 536 genes) presenting *R*(*ω*)<1 in *Buchnera sp.* and *Blochmannia sp.* endosymbiont genomes, respectively (Figure 1A and 1B and Table S1). When we examined the constraints operating at synonymous and non-synonymous sites at these genes and compared them to those in the set of genes with *R (ω)>1*, we noticed that increased selection intensity was partially due to more relaxed constraints at synonymous sites, but mainly to significantly stronger constraints at non-synonymous sites in this dataset (Table 1). For example, the increase factor of *d_N_* [*R(d_N_)*] when comparing endosymbionts with free-living bacteria in the dataset with *R*(*ω*)<1 was half that in the dataset of genes showing *R(ω)>1*. In summary, increments of *ω* in endosymbiotic bacteria are negatively correlated with increments in synonymous substitutions and positively correlated with non-synonymous substitutions increments for *Buchnera* (Perason's correlation; *ρ*_R(*ω*)-R(dS)_ = −0.696, *P*≪10^−12^, and *ρ*_R(*ω*)-R(dN)_ = 0.539, *P*≪10^−12^) and *Blochmannia* (*ρ*_R(*ω*)-R(dS)_ = −0.540, P≪10^−12^, and *ρ*_R(*ω*)-R(dN)_ = −0.421, P≪10^−9^).

### Differential Functional Enrichment in Highly Constrained Genes in Endosymbiontic Bacteria

**Figure 2:**
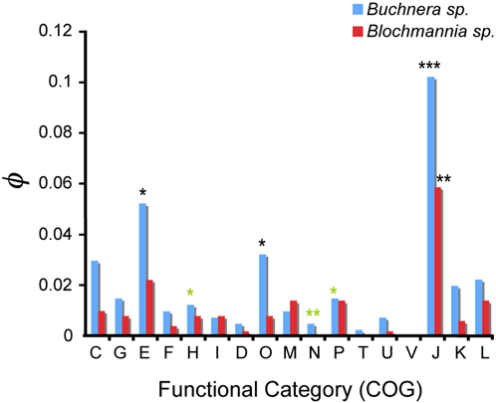
Distribution of highly constrained genes among the functional categories in *Buchnera sp.* (blue bars) and *Blochmannia sp.* (red bars). The different functional categories as explained by the Cluster of Orthologous Groups (COG) are represented in the X-axis. The height of the bar represents the relative contribution of each class (*i*) of size (*t*), to the total number of genes under strong selective constraints (*n_i_*: R(*ω*) = *ω_e_*/*ω_f_*<1) when considering the whole dataset (*T*). This normalized number hence was calculated as *φ* = (*n_i_*/*t*) * (*t*/*T*). Classes showing significant enrichment with highly constrained genes under a hypergeometric distribution are labeled by (*, *P*<0.05; **, *P*<10^−2^; ***, *P*<10^−3^). We also labeled those functional classes significantly underrepresented by highly constrained genes using green stars.

To test the link between the biological and evolutionary characteristics *of Buchnera* and *Blochmannia* and the constraints on their genomes we analyzed the distribution of genes with *R*(ω)<1 among the different functional classes obtained using COG terms. We examined metabolism (represented by 161 genes and 229 genes in *Buchnera* and *Blochmannia*, respectively), cellular processes and signaling (represented by 99 and 108 genes in *Buchnera* and *Blochmannia*, respectively) and information storage and processing (represented by 127 and 153 genes in *Buchnera* and *Blochmannia*, respectively). We discarded genes that were ambiguously classified. The total number of genes, number of genes with *R*(*ω*)<1 and enrichment of each functional sub-category are indicated in Table 2. We tested the significance of the enrichment with genes highly conserved in endosymbionts compared to their free-living relatives using the hypergeometric distribution as explained in material and methods. Several of the functional categories examined presented a high percentage of constrained genes in both *Buchnera* and *Blochmannia*, although this was more pronounced in *Buchnera* than in *Blochmannia* (Figure 2). *Buchnera* presented several of the categories enriched with genes under stronger constraints than in its free-living relatives, including genes involved in transport and metabolism of essential amino acids (category E); in post-translational modification and chaperones (O); and in translation, ribosomal structure and biogenesis (J) (Figure 2). *Blochmannia* only presented evidence for such enrichment in the category of genes involved in translation, ribosomal structure and biogenesis. Several other functional categories presented poor percentages (significantly low) of strongly constrained genes in *Buchnera* but not in *Blochmannia* including the categories of coenzyme transport and metabolism, cell motility, and inorganic ion transport and metabolism (Figure 2). Other categories such as those including defense genes (V), signal transduction (T), etc. comprised a very low number of genes and hence presented no statistical power for rejecting the null hypothesis of no differential enrichment with constrained genes.

**Table 2:** Distribution of constrained genes in endosymbiotic bacteria of aphids (Buch) and carpenter ants (Bloc) among the functional categories classified using COG.

Category	Sub-category	#Genes		# Genes R(ω)<1		%Genes	
		Buch	Bloc	Buch	Bloc	Buch	Bloc
Met	C	40	42	12	5	30.0	11.9
	G	19	28	6	4	31.6	14.3
	E	47	58	21	11	44.7	18.9
	F	20	23	4	2	20.0	8.7
	H	25	34	5	4	20.0	11.8
	I	10	25	3	4	33.0	16.0
	P	12	19	6	7	50.0	36.8
CPS	D	8	13	2	1	25.0	7.8
	O	33	28	13	4	39.4	14.3
	M	17	48	4	7	23.5	14.6
	N	23	0	2	0	8.7	0.0
	T	4	4	1	0	25.0	0.0
	U	10	13	3	1	33.0	7.7
	V	3	2	0	0	0.0	0.0
ISP	J	82	106	41	29	50.0	27.4
	K	14	16	8	3	57.0	18.8
	L	31	31	9	7	29.0	22.6

### Heterogeneous Functional Divergence among Metabolic Pathways in Endosymbionts

**Figure 3:**
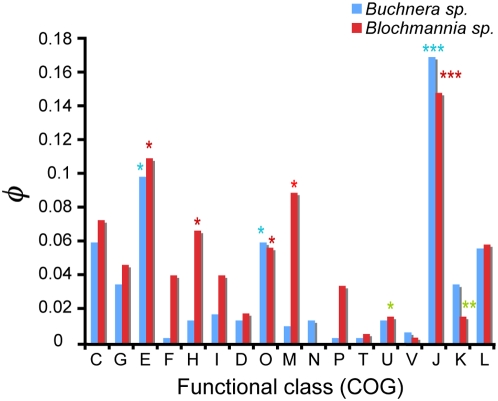
Distribution of genes under functional divergence among the functional categories in *Buchnera sp.* (blue bars) and *Blochmannia sp.* (red bars). The different functional categories as explained by the Cluster of Orthologous Groups (COG) are represented in the X-axis. The height of the bar represents the relative contribution of each class (*i*) of size (*t*), to the total number of genes under functional divergence (*n_i_*: R(*ω*) = *ω_e_*/*ω_f_*<1) when considering the whole dataset (*T*). This normalized number hence was calculated as *φ* = (*n_i_*/*t*) * (*t*/*T*). Classes showing significant enrichment with genes under functional divergence under a hypergeometric distribution are labeled by (*, *P*<0.05; **, *P*<10^−2^; ***, *P*<10^−3^). We also labeled those functional classes significantly underrepresented by highly constrained genes using green stars.

Based on the assumption that endosymbiosis involved a dramatic biological leap made possible by functional shifts in pre-existing proteins, we tested for the presence of functional divergence in *Buchnera* and *Blochmannia*. Even though both endosymbiotic systems share common biochemical traits (for example, the need for essential amino acids in their diet as well as nitrogen compounds), they also possess distinct biological requirements. For example, unlike aphids, ants are unable to fix and reduce sulphur, which is provided by the endosymbiont. We attempted to test whether analyses of functional divergence could shed light on the connection between protein variability and biochemical host-endosymbiont links. Our test identified 63.7% and 78.6% of genes to be under functional divergence in *Buchnera* and *Blochmannia*, respectively. *Buchnera* presented three functional categories enriched with functional divergence, including the one involved in amino acid transport and metabolism (E), post-translational modification and chaperones (O) and translation, ribosomal structure and biogenesis (J) (Figure 3). *Blochmannia* also showed significant evidence of functional divergence enrichment at these categories and in additional categories involved in coenzyme transport and metabolism (H), and cell wall and membrane biogenesis (M) (Figure 3). Other categories in *Blochmannia* presented evidence of being poorly populated by genes under functional divergence including that comprising genes involved in intra-cellular trafficking (U) and transcription (K) (Figure 3).

### Functional Divergence in the Endosymbiotic Metabolic Pathways

**Figure 4:**
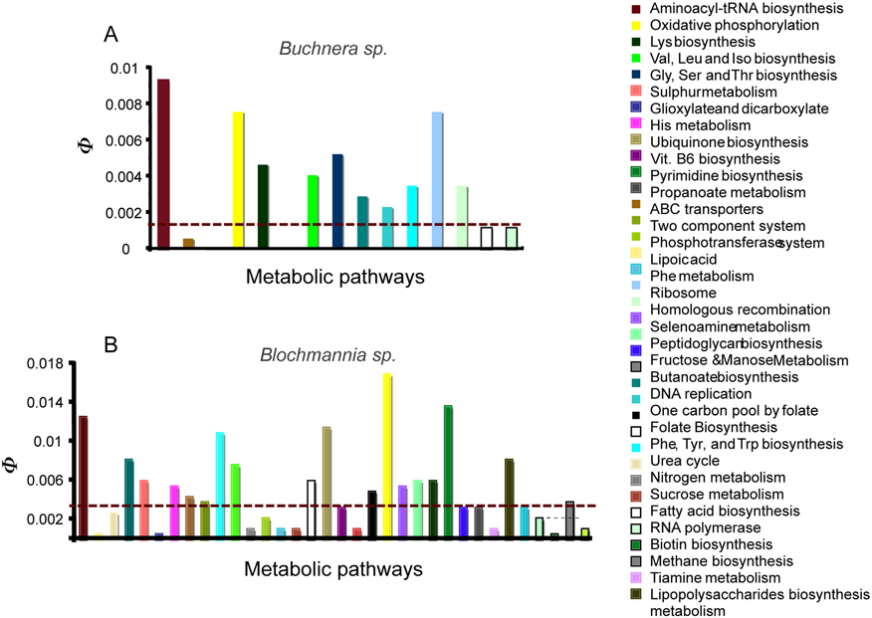
Distribution of genes under functional divergence among the metabolic pathways significantly enriched or impoverished with these genes in *Buchnera* (A) and *Blochmannia* (B). The different metabolic classes are color-coded. Dotted line separates metabolic pathways enriched with functionally divergent genes (above the line) from those impoverished with these genes (below the line). The height of the bar represents the relative contribution of each class (*i*) of size (*t*), to the total number of genes under functional divergence (*n_i_*: R(*ω*) = *ω_e_*/*ω_f_*<1) when considering the whole dataset (*T*). This normalized number hence was calculated as *φ* = (*n_i_*/*t*) * (*t*/*T*).

To identify the relationship between functional divergence and endosymbiosis we analyzed the distribution of genes between the different metabolic pathways and tested the enrichment of pathways with genes under functional divergence using the hypergeometric distribution. We identified and classified genes into 67 different pathways. In *Buchnera* symbionts we found 10 pathways to be significantly enriched and 4 to be significantly impoverished with proteins that underwent functional divergence after symbiosis (Figure 4A and Table S2). Among the enriched pathways we identified those including proteins involved in the biosynthesis of aminoacyl-tRNA of the 10 essential amino acids needed by the aphid, biosynthesis of the essential amino acids (Lysine, Valine, Leucine, Isoleucine, Glycine, Serine, Threonine, Phenylalanine, Tyrosine and Tryptophan), DNA replication, ribosomes, and homologous recombination. ABC transporters, two-component system, phosphotransferases and RNA polymerase were the metabolic pathways showing the least number of functionally divergent genes. In the case of *Blochmannia* we could identify and classify genes into 71 different pathways. 506 genes showed evidence of functional divergence and because of this large number we applied a chi-square distribution to test for enrichment with functional divergence. This test was performed so that the chi-square value was calculated for each metabolic class (pathways) as follows:
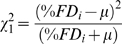



Here *%FD_i_* stands for the proportion of the genes in that metabolic class *i* showing functional divergence, while *μ* is the mean proportion of genes under functional divergence throughout the metabolic pathways. Analyses of *Blochmannia* pathways identified similar pathways as those in *Buchnera sp.* to be enriched with genes under functional divergence, including aminoacyl-tRNA for essential amino acids for the host, DNA replication, essential amino acids biosynthesis, folate biosynthesis and oxidative phosphorylation (Figure 4B). The pathways for ABC transporters, phosphotransferases, and the two-component system were also impoverished with genes under functional divergence. However, in contrast to *Buchnera* 18 pathways were enriched and 16 pathways impoverished for genes under functional divergence. For example, among enriched pathways with proteins under functional divergence not present in *Buchnera* were those involved in the metabolism of sulphur, histidine, vitamine B6, selenamine acid, pyrimidine; biosynthesis of liposaccharides, ubiquinones, fatty acids, and peptidoglycans, and the pathway of RNA polymerase. In contrast to *Buchnera* other pathways were impoverished for proteins under functional divergence, including those involved in metabolism of nitrogen, urea, phenylalanine, starch and sucrose, galactose, fructose and mannose, propanoate, thiamine, biotine, methane and butanoate (Figure 4B and Table S2).

## Discussion

Symbiosis, which has been generally the result of the association of organisms with different biological complexities, has been regarded as central to the rapid adaptive radiation [19],[20],[22]. These different metabolic complexities translate into dramatic lifestyle changes in one or both partners of the relationship that are instrumental to their successful metabolic marriage. In attempt to understand this metabolic communication and the key evolutionary events having led to them we have addressed a fundamental question regarding endocellular symbiosis: What are the main genome evolutionary events that enabled the functional/metabolic communication between symbiotic bacteria and insects? In some cases, such as in endosymbiotic bacteria of aphids, hints about the complexity of this communication have been provided through genetic, evolutionary and metabolic studies. Other associations, such as the one established between ants and their symbionts remain intriguing because of the apparent balance of the insect's diet. Genomes sequencing projects of endosymbiontic bacteria of insects offer an unprecedented opportunity for establishing the biological and evolutionary bases of endosymbiosis in insects. However, presently we rely on a handful of endosymbiotic genomes to infer general results. Inference of convergent dynamics is only possible if the different endosymbiotic bacteria examined are not directly related from the phylogenetic perspective such as in the case of *Buchnera* and *Blochmannia* endosymbionts [41]–[44]. In addition, unlike aphids, ants present a balanced diet [45] and endosymbionts have been shown to be essential only during the pupation phase [46]. The presence of several full genomes for ants and aphids endosymbionts and the ecological differences between aphids and ants makes it possible to identify evolutionary genome dynamics that are either strictly related to the establishment of symbiosis or to the ecological capabilities provided by the bacterium to the host.

Most of the studies aimed at identifying signs of adaptive evolution in protein-coding genes are built on the Neo-Darwinists theoretical ground, which is based on deterministic models that assume infinite populations sizes. These theories and studies dismissed the idea of genetic drift following the rationale that most of the observed polymorphism is maintained in the population through balancing selection (for review see [47]). In the light of the neutral theory of molecular evolution however, most of the variation is selectively neutral and maintained by genetic drift. This rather than the exception is a frequent observation in organisms' populations that in fact are responsible for generating a huge ecological and biological diversity such as the case of endosymbiotic bacteria of insects. Our genome-wide evolutionary analyses unravel the clear effect of genetic drift in both endosymbiotic systems studied. This is also supported by the fact that non-synonymous sites are much more relaxed than synonymous sites in endosymbionts because these sites are under selection in free-living bacteria. Strikingly however, *Blochmannia* presents more relaxed constraints than *Buchnera* (for example the percentage of genes with *R(ω)*<1 in *Buchnera* is nearly twice as much as that in *Blochmannia*) probably because of its limited role during the lifecycle of the ant host. The more relaxed constraints in the the greater genome size of *Blochmannia* pinpoint the existence of non-functionalized genes probably due to the younger symbiosis of *Blochmannia* with ants (around 70 MYA for *Blochmannia* against the approximately 200 MYA for *Buchnera*) [48].

The next question we asked was whether relaxed constraints were random (as a result of genetic drift) or they correlated with the requirements of the host and the bacterium. Our functional class enrichment analyses indicate that, in accordance with the expectation given the metabolic requirements of the host, genes of transport and metabolism of essential amino acids, ribosome structures and translation and genes involved in posttranslational modifications and chaperones were more constrained in *Buchnera* than in their free-relative bacterial homologs. Genetic drift in *Buchnera* has been extensively demonstrated (for example see [27]). Conserved constraints at non-synonymous sites can therefore be due either to a major need for these proteins to perform their ancestral functions or alternatively to their functional divergence to perform different but more important functions in the endosymbionts. Indeed, all the functional categories showing *R*(*ω*)<1 have been previously reported to play key roles in host's ecology (for example supplementing host's diet with essential amino acids [49],[50]). Furthermore, the high evolutionary rates of genes of ribosomal structure and translational proteins, posttranslational modification and folding supports their functional divergence since *Buchnera* accumulates slightly deleterious mutations at high rates at these proteins, being unlikely to conserve their ancestral function. Chaperones have been reported to improve their folding activity, probably by functional divergence, thereby buffering the effects of Muller's ratchet [51],[52]. Unlike the transport and metabolism pathways, we detected significant enrichment of genes under strong selective constraints in the classes of ribosomal structures and translation from *Blochmannia*, which may be in accordance with ants being omnivorous [53]–[57].

We show evidence that in small effective population sizes of endosymbionts genetic drift may be followed by increase of their effective population sizes where selection can become efficient in filtering the mutations fixed enabling fixation of slightly advantageous mutations (For a discussion on the subject see [47]). Hence, slightly advantageous mutations are likely to be fixed neutrally in the population possibly leading to changes in proteins' functions (for example to functional divergence). Functional divergence is based on the assumption that changes in the evolutionary conservation of certain protein residues may lead to a change in function. Following this idea many groups have developed statistical methods to identify functional divergence after gene duplication (for example see [58]–[62]), although these methods were not developed for genome wide analyses. As in gene duplication, changing lifestyles of organisms may lead to gene redundancies in the new environments. Consequently, the selective pressures to delete such redundancies decreases allowing the accumulation of mutations with slight effects on fitness and hence for functional divergence. Here we developed a novel method to identify genome wide functional divergence that is in theory applicable to any organism with changing lifestyles, such as pathogens, extremophiles, etc. The results correlated strongly with the ecological requirements of the hosts. Interestingly, we found that pathways involved in tRNA synthesis, of the 10 essential amino acids, the metabolism of these 10 essential amino acids, DNA replication, ribosomes, and homologous recombination are highly enriched with genes that show evidence of functional divergence in both endosymbiotic systems. This convergence may be due to the possible unbalanced diet of ants during the pupation phase. Functional divergence of genes involved in DNA replication, including helicase (DnaB), primase (DnaG) and the SSB protein in *Buchnera* may make replication dependent upon the population of the host. Further, other metabolic pathways such as ABC transporters, phosphotransferases and the two-component system were poorly populated by genes under functional divergence. These genes are probably involved in the transport of proteins and ions from the bacterium to the host, and are therefore under strong functional constraint. Such seems the case also of the RNA polymerase category. The fact that *Buchnera* and *Blochmannia* both present genes evolving under different constraints in the replication pathway, in addition to the lack of genes in *Blochmannia* involved in initiating replication (*dnaA*, *priA* and *recA*) [63] may support the need of a slowing of the bacterial replication in a host-controlled way.

Other categories in *Blochmannia*, but not in *Buchnera*, presented enrichment with genes under functional divergence including categories with genes involved in metabolism of sulphur, histidine, lipopolysaccharides, fatty acids, peptidoglycans, and nitrogen. All these categories include genes that are essential to provide the host with the ability to reduce sulphur, and to recycle nitrogen through the endosymbiont urease, as previously suggested [64]. Other enriched categories were polysaccharides and peptidoglycans that are essential components of the cell wall (specifically of the outer membrane) and they provide a rather more structured membrane to the bacterium rendering it more resistant [63] to the hostile environment of the cytosol [65]. Finally, metabolic pathways related to the metabolism of sugars (for example, Fructose) are highly impoverished with genes under functional divergence probably due to the need to preserve ancestral functions so as to deal with the sugar-rich ant diet. In conclusion, metabolic pathways related to host ecological requirements are either enriched or impoverished with genes under functional divergence, both extremes ensuring the conservation of the ancestral optimized function or specializing the pathway for the overproduction or improvement of the final substrate. In summary, we show a new way of testing for functional divergence that can be potentially applied to other biological systems such as pathogens and organisms living in different and extreme environments. Using this method we provide evidence of the main underlying evolutionary mechanisms that were essential for the establishment of endosymbiosis and for the specific metabolic communication between the bacterium and the insect host. The differing evolutionary dynamics of the metabolic pathways and their correlation with the insect's ecological requirements reveal a clear link between bacterial symbiosis and species ecological innovation.

## Material and Methods

### Genomes and Alignments

We provide the full list of genes examined for functional divergence in table S1. In our analysis we used the four genomes of the endosymbiotic bacterium of aphids *Buchnera aphidicola*, including strains *Acyrthosiphon pisum* (*BAp*: NC_002528), *Schizaphis graminum* (*BSg*: NC_004061), *Baizongia pistaciae* (*BBp*: NC_004545) and *Cinara cedri* (*BCc*: NC_008513). For he same genes we used the genomes of the free-living relatives *Escherichia coli* K12 (*Ec*: NC_000913); *Salmonella tyhimurium* (*St*: NC_003197); *Shigella flexneri* (*Sf*: NC_004741); and *Erwinia carotovora* (*Eca*: NC_004547). For the analysis of functional divergence we used the external (outgroup) genome of *Photorhabdus luminescens* (*Pl*: NC_005126), due to its appropriate phylogenetic proximity to both groups of bacteria. In the case of the endosymbiotic bacteria of carpenter ants, we used *Candidatus Blochmannia floridanus* (*Bf*: NC_005061) and *Candidatus Blochmannia pennsylvanicus* (*Bp*: NC_007292), the only two genomes available and fully sequenced. These two endosymbiotic systems present the possibility of generalizing our conclusions because both have independent evolutionary origins [41] and yet they are phylogenetically close to the same free-living bacterial relatives.

With each one of the genes in the genomes we performed Blast searches to find the orthologs in the other genomes, considering acceptable only those genes showing reciprocal best top hits with scores of less or equal than 10^−4^. For each one of the genes we built multiple protein alignments using ClustalW program with the default parameters [66]. Then we obtained the multiple alignments for protein-coding sequences concatenating nucleotide triples according to the corresponding protein alignment. All multiple sequence alignments were carefully inspected before proceeding with the evolutionary analyses.

### Characterisation of Selective Constraints in Endosymbiotic Genomes

Protein functional divergence along a phylogenetic lineage requires a re-distribution of evolutionary rates along a protein and the rapid fixation of functionally advantageous mutations through episodic (punctual) Darwinian selection in that lineage. The second condition to be met to consider a mutational event as responsible for functional divergence is that the mutation became fixed under strong purifying selection after speciation post-dating that lineage. This involves an increase in the number of amino acid replacing nucleotide substitutions in the lineage leading to that cluster while synonymous substitutions remain neutral. Consequently, as a result of functional divergence we expect an increase of the non-synonymous-to-synonymous rates ratio (ω = *d_N_*/*d_S_*), which has been used in numerous studies as an indicator of the force of selection acting on protein-coding genes (see for example [51],[67],[68]). While the number of non-synonymous nucleotide substitutions per site (*d_N_*) is under selection because they involve changing the amino acid composition of sequences, synonymous substitution per site (*d_S_*) accumulate neutrally due to their silent effect on protein's amino acid composition. However synonymous sites may also be under selection caused by translational efficiency or stability of RNA molecules [69]–[72]. Assuming however that synonymous sites evolve neutrally, Values of *ω*<1 indicates that most of the amino acid substitutions are deleterious and removed by selection (purifying selection); *ω* = 1 indicates neutral evolution, while *ω*>1 provides evidence for the fixation of amino acid replacing mutations by positive selection.

In this study we analyzed whether lineages of endosymbiotic bacteria of insect show evidence of functional divergence by presenting different ω values than expected under linear evolution. Functional divergence involves a shift in the selection forces acting on amino acid sites of protein-coding genes. Therefore, irrespective of the constraints on synonymous sites, endosymbiotic ω (*ω_e_*) will yield similar values to those in their free-living relatives (*ω_f_*) if the constraints were the same in both groups of bacteria and different values if the selective constraints changed in one clade compared to the other. To characterise the changes in selective constraints in the endosymbiotic lineage we estimated *d_N_* and *d_S_* for the endosymbiotic lineage and free-living bacterial lineage using the program YN00 from the PAML package version 4.0 [73]. We studied the full genome of *Buchnera sp.* and *Blochmannia sp.* including a total of 509 and 536 genes, respectively. We estimated the number of substitutions per site using the modified method of Nei and Gojobori [74] as implemented in YN00. Then, we performed comparisons of the selective constraints in each gene between endosymbionts and their free-living cousins by dividing their corresponding ω values (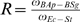). To increase the coherence of the comparison analyses between both symbiotic systems, we estimated nucleotide substitutions for the genes in the comparisons of each one of the endosymbiotic lineages (*BAp*-*BSg*, *Bf*-*Bp*) to their free-living relatives *Ec*-*St*. We performed these comparisons because these pairs show similar divergence times (50–100 Million Years; [32],[75]).

In this manuscript we do not deal with the causes of relaxed constraints in symbiotic bacteria. For example, synonymous sites may be under relaxed constraints in genes that were over-expressed in free-living bacteria but are no longer so in endosymbionts. The effect of translational efficiency in selection over synonymous sites has been previously shown [76]. Further, we have recently reported evidence of selection on non-synonymous sites shaped by translational robustness [77]. However, irrespective of the reasons for such constraints the aim was to show that in fact ω was affected by variation of such constraints at synonymous and non-synonymous sites but to a different extent.

### Identification of Functional Divergence

In this manuscript we identified functional divergence type I as described previously [59]. Functional divergence type I involves the change in the selection constraints at specific amino acid sites of a protein in a phylogenetic cluster in comparison to another. The question we asked here is what genes have dramatically changed their selective constraints during the evolution of endosymbiosis in comparison with their free-living bacterial relatives, indicating hence a change in function. The test performed here is therefore unidirectional (1 tail test). In particular, we wanted to examine the acquisition of functional importance at amino acid sites in endosymbiotic proteins that were evolving neutrally in their free-living cousins (indicating functional divergence). In statistical and evolutionary terms, the purpose was to identify amino acid sites that were variable at free-living bacteria, but that, after they have undergone important physicochemical changes in the lineage leading to endosymbionts, became highly constrained (conserved) in the lineages postdating endosymbionts speciation events. Because endosymbiotic bacteria have been evolving under genetic drift, we expect sites to be more variable than in their free-living relatives and hence positive identification of sites under functional divergence would provide a conservative measure of such selection constraints shifts.

**Figure 5:**
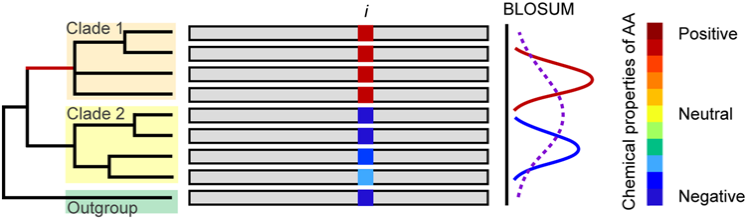
Genome wide identification of functional divergence. Proteins are identified to be under functional divergence if they show amino acid sites presenting significant evidence of shifts in the evolutionary rates in cluster 1 (cluster under study) compared to cluster 2 (background cluster). To measure functional divergence at site *i*, we first calculate all pair-wise BLOSUM transition values in the pair-wise comparison of the sequences in the tree. Sequences in cluster 1 are compared to the outgroup and the BLOSUM transition values between outgroup and cluster 1 generate a distribution that is compared to that generated when comparing sequences of cluster 2 to the outgroup. The change in the physico-chemical properties of amino acids from cluster 1 to cluster is indicated by colored squares. If the transition scores are significantly more radical when comparing the outgroup to cluster 1 at that amino acid site of the protein than when we compared the outgroup to cluster 2 then we consider the site to be under functional divergence. The significance of the transition scores in cluster 1 is calculated by comparing the distribution of scores in cluster 1 to that in cluster 2 and significance is considered at the 1% confidence level.

To conduct a genome wide analysis of functional divergence, we developed a fast, accurate, and simple statistical method to identify functional divergence in genomic data (Figure 5 shows the different steps of the method in a schematic way). Bayesian and maximum-likelihood approaches developed previously to identify functional divergence (for example, [58],[59]) could not be used in this study because: i) such methods have been implemented for the analysis of single genes; ii) they have been devised to identify functional divergence after gene duplication; and iii) they require at least four sequences per clade to conduct the analysis. These requirements are not always met and in the case of endosymbionts of ants only two genome sequences have been fully sequenced. Our method uses BLOSUM scores to compare the evolutionary distance between two clades of homologous proteins and an outgroup sequence, providing a fast and conservative way of identifying amino acid sites under functional divergence. The input is a protein sequence alignment of the two pre-defined clades and an outgroup sequence. The endosymbiont clade was defined as the clade-of-interest (which we call clade 1), so that the method identifies sites in that clade which have diverged significantly further in function from the outgroup sequence than have the homologous sites in the second clade (clade 2) (see Figure 5 for details).

For each column in the alignment, we calculate the BLOSUM scores for the substitution between each amino acid in each clade and the outgroup residue. Since the probability of observing an unlikely substitution increases with the divergence time between sequences, each pairwise BLOSUM score is divided by the Poisson distance between the sequences from which the two residues are derived – that is, the outgroup and one other sequence. Even though amino acid substitutions are under selective constraints, we assume that some sites may evolve neutrally and some others under constraints but that these effects cancel each other out when averaged along the sequence. We then calculate the mean BLOSUM score between all clade 1 residues and the outgroup (clade 1 mean: 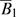), all clade 2 residues and the outgroup (clade 2 mean 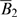), and the standard error of both these quantities (*SE_1_*, *SE_2_*). Negative BLOSUM scores indicate rarely observed substitutions, while positive scores indicate commonly observed ones. Since we are attempting to identify sites in clade 1 that are under functional divergence when compared to clade 2, we filter out all sites for which the value of clade 1 mean is positive (indicating hence conservative substitutions; 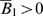), and also those sites for which the value of clade 2 mean is negative. Further, to avoid obtaining spurious results due to the high genetic drift experienced by endosymbiotic bacteria, we filter out all sites that are not completely conserved in clade 1. Finally, we calculate a Z-score for the column to estimate the probability of the observed putative functionally divergent site.

### Metabolic Data

The database KEGG (Kyoto Encyclopedia of Genes and Genomes) [78] links genomic information with current knowledge on functional information. It consists of four main sections including pathway information, genes collections from all fully sequenced genome, chemical information (for example, cell compounds, enzymes, drugs approved etc.) and relationships of various biological objects. It also integrates a number of software that link all the knowledge about the pathway, comprising information genes present in a particular pathway for a specific species.

We downloaded the file genes_pathway.list from KEGG ftp site (ftp://ftp.genome.jp/pub/kegg/linkdb/genes/), which contains all links between genes in KEGG database, and the pathway in which the gene is present. To determine the possible link between the interaction of the endosymbiont metabolism and that of the host, we tested if particular pathways showed evidence of proteins under functional divergence constraints in the endosymbiotic lineages.

The functions of the genes were determined using Cluster of Orthologous Groups (COG) [79]. We had genes from 17 different sub-categories and they were; Translation, ribosomal structure and biogenesis (J), Transcription (K), and Replication, recombination and repair (L) from Information Storage and Processing (ISP). Cell cycle control, cell division, chromosome partitioning (D), Defense mechanisms (V), Signal transduction mechanisms (T), Cell wall/membrane/envelope biogenesis (M), Cell motility (N), Intracellular trafficking, secretion, and vesicular transport (U), Posttranslational modification, protein turnover, chaperones (O) from Cellular Processes and Signaling (CPS). Energy production and conversion (C), Carbohydrate transport and metabolism (G), Amino acid transport and metabolism (E), Nucleotide transport and metabolism (F), Coenzyme transport and metabolism (H), Lipid transport and metabolism (I), and Inorganic ion transport and metabolism (P) from Metabolism (Met).

### Statistical Analyses

The main tests we performed were those aimed at determining whether any of the functional classes determined using Cluster of Orthologous Groups (COG) [79] or any of the pathways presented evidence of enrichment with genes under strong selective constraints and/or functional divergence. Since the number of data was finite per class and we were using discrete number of genes we conducted our tests using an approximation to the exact Fisher's test, called Hypergeometric approximation. Under the Hypergeometric density function, the probability of observing *K* events in the class *m*, from a sample size of *N* is:
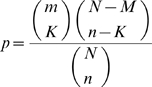



This probability density accounts for the unequal size of the different classes and for non-normal distribution of data, making the test statistically robust to deviations from normality due to finite data sets. We used this test to identify functional classes and metabolic pathways enriched or impoverished by genes under functional divergence.

## Supporting Information

Table S1 The ratio between the intensities of selection in the endosymbiont *Buchnera aphidicola* genomes and *Blochmannia sp.* and their free-living cousins. Genes are ordered alfabeticaly according to gene name in *E. coli*. Data missing or that could not be estimated are indicated by -.
(0.70 MB DOC)

Table S2 Functional divergence analysis in the metabolic pathways of *Buchnera* and *Blochmannia* endosymbionts.
(0.09 MB DOC)

## References

[1] Messier W, Stewart CB (1997). Episodic adaptive evolution of primate lysozymes.. Nature.

[2] Gould SJ, Eldredge N (1993). Punctuated equilibrium comes of age.. Nature.

[3] Drummond DA, Bloom JD, Adami C, Wilke CO, Arnold FH (2005). Why highly expressed proteins evolve slowly.. Proc Natl Acad Sci U S A.

[4] Bloom JD, Raval A, Wilke CO (2007). Thermodynamics of neutral protein evolution.. Genetics.

[5] Lin YS, Hsu WL, Hwang JK, Li WH (2007). Proportion of solvent-exposed amino acids in a protein and rate of protein evolution.. Mol Biol Evol.

[6] Gaucher EA, Gu X, Miyamoto MM, Benner SA (2002). Predicting functional divergence in protein evolution by site-specific rate shifts.. Trends Biochem Sci.

[7] Lopez P, Casane D, Philippe H (2002). Heterotachy, an important process of protein evolution.. Mol Biol Evol.

[8] Abhiman S, Sonnhammer EL (2005). Large-scale prediction of function shift in protein families with a focus on enzymatic function.. Proteins.

[9] Gu J, Neary JL, Sanchez M, Yu J, Lilburn TG (2007). Genome evolution and functional divergence in Yersinia.. J Exp Zool B Mol Dev Evol.

[10] Fitch WM, Markowitz E (1970). An improved method for determining codon variability in a gene and its application to the rate of fixation of mutations in evolution.. Biochem Genet.

[11] Ohno S (1970). Evolution by Gene Duplication.

[12] Li WH, Gojobori T (1983). Rapid evolution of goat and sheep globin genes following gene duplication.. Mol Biol Evol.

[13] Clark AG (1994). Invasion and maintenance of a gene duplication.. Proc Natl Acad Sci U S A.

[14] Hughes AL (1994). The evolution of functionally novel proteins after gene duplication.. Proc Biol Sci.

[15] Fryxell KJ (1996). The coevolution of gene family trees.. Trends Genet.

[16] Nei M, Gu X, Sitnikova T (1997). Evolution by the birth-and-death process in multigene families of the vertebrate immune system.. Proc Natl Acad Sci U S A.

[17] Force A, Lynch M, Pickett FB, Amores A, Yan YL (1999). Preservation of duplicate genes by complementary, degenerative mutations.. Genetics.

[18] Gu X (2003). Evolution of duplicate genes versus genetic robustness against null mutations.. Trends Genet.

[19] Schluter D (2000). The ecology of adaptive radiation.

[20] Rundle HD, Nosil P (2005). Ecological speciation.. Ecol Lett.

[21] Johnson SD, Linder HP, Steiner KE (1998). Phylogeny and radiation of pollination systems in DISA (Orchidaceae).. Am J Bot.

[22] Levin DA (2006). Flowering phenology in relation to adaptive radiation.. Syst Bot.

[23] Price PW, Margulis L, Fester R (1991). The web of life: development of over 3.8 billion years of trophic relationships.. Symbiosis as a Source of Evolutionary Innovation: Speciation and Morphogenesis.

[24] Andersson SG, Kurland CG (1998). Reductive evolution of resident genomes.. Trends Microbiol.

[25] Perez-Brocal V, Gil R, Ramos S, Lamelas A, Postigo M (2006). A small microbial genome: the end of a long symbiotic relationship?. Science.

[26] Toft C, Fares MA (2008). The evolution of the flagellar assembly pathway in endosymbiotic bacterial genomes.. Mol Biol Evol.

[27] Moran NA (1996). Accelerated evolution and Muller's rachet in endosymbiotic bacteria.. Proc Natl Acad Sci U S A.

[28] Lynch M (1997). Mutation accumulation in nuclear, organelle, and prokaryotic transfer RNA genes.. Mol Biol Evol.

[29] Lynch M (1996). Mutation accumulation in transfer RNAs: molecular evidence for Muller's ratchet in mitochondrial genomes.. Mol Biol Evol.

[30] Rispe C, Moran NA (2000). Accumulation of deleterious mutations in endosymbionts: Muller's ratchet with two levels of selection.. Am Nat.

[31] Brynnel EU, Kurland CG, Moran NA, Andersson SG (1998). Evolutionary rates for tuf genes in endosymbionts of aphids.. Mol Biol Evol.

[32] Clark MA, Moran NA, Baumann P (1999). Sequence evolution in bacterial endosymbionts having extreme base compositions.. Mol Biol Evol.

[33] Funk DJ, Wernegreen JJ, Moran NA (2001). Intraspecific variation in symbiont genomes: bottlenecks and the aphid-buchnera association.. Genetics.

[34] Wernegreen JJ, Moran NA (2000). Decay of mutualistic potential in aphid endosymbionts through silencing of biosynthetic loci: Buchnera of Diuraphis.. Proc Biol Sci.

[35] Gil R, Sabater-Munoz B, Latorre A, Silva FJ, Moya A (2002). Extreme genome reduction in Buchnera spp.: toward the minimal genome needed for symbiotic life.. Proc Natl Acad Sci U S A.

[36] Abbot P, Moran NA (2002). Extremely low levels of genetic polymorphism in endosymbionts (Buchnera) of aphids (Pemphigus).. Mol Ecol.

[37] Lambert JD, Moran NA (1998). Deleterious mutations destabilize ribosomal RNA in endosymbiotic bacteria.. Proc Natl Acad Sci U S A.

[38] van Ham RC, Kamerbeek J, Palacios C, Rausell C, Abascal F (2003). Reductive genome evolution in Buchnera aphidicola.. Proc Natl Acad Sci U S A.

[39] Maezawa K, Shigenobu S, Taniguchi H, Kubo T, Aizawa S (2006). Hundreds of flagellar basal bodies cover the cell surface of the endosymbiotic bacterium Buchnera aphidicola sp. strain APS.. J Bacteriol.

[40] Shigenobu S, Watanabe H, Hattori M, Sakaki Y, Ishikawa H (2000). Genome sequence of the endocellular bacterial symbiont of aphids Buchnera sp. APS.. Nature.

[41] Herbeck JT, Degnan PH, Wernegreen JJ (2005). Nonhomogeneous model of sequence evolution indicates independent origins of primary endosymbionts within the enterobacteriales (gamma-Proteobacteria).. Mol Biol Evol.

[42] Dasch GA, Weiss E, Chang KP, Krieg NR, Holt JG (1984). Endosymbionts of insects.. Bergey's Manual of Systematic Bacteriology.

[43] Schroder D, Deppisch H, Obermayer M, Krohne G, Stackebrandt E (1996). Intracellular endosymbiotic bacteria of Camponotus species (carpenter ants): systematics, evolution and ultrastructural characterization.. Mol Microbiol.

[44] Sameshima S, Hasegawa E, Kitade O, Minaka N, Matsumoto T (1999). Phylogenetic comparison of endosymbionts with their host ants based on molecular evidence.. Zool Sci.

[45] Pfeiffer M, Linsenmair KE (2000). Contributions to the life history of the Malaysian giant ant Camponotus gigas (Hymenoptera, Formicidae).. Insectes Sociaux.

[46] Zientz E, Beyaert I, Gross R, Feldhaar H (2006). Relevance of the endosymbiosis of Blochmannia floridanus and carpenter ants at different stages of the life cycle of the host.. Appl Environ Microbiol.

[47] Hughes AL (2007). Looking for Darwin in all the wrong places: the misguided quest for positive selection at the nucleotide sequence level.. Heredity.

[48] Sauer C, Stackebrandt E, Gadau J, Holldobler B, Gross R (2000). Systematic relationships and cospeciation of bacterial endosymbionts and their carpenter ant host species: proposal of the new taxon Candidatus Blochmannia gen. nov.. Int J Syst Evol Microbiol.

[49] Douglas AE (1998). Nutritional interactions in insect-microbial symbioses: aphids and their symbiotic bacteria Buchnera.. Annu Rev Entomol.

[50] Sandstrom J, Telang A, Moran NA (2000). Nutritional enhancement of host plants by aphids - a comparison of three aphid species on grasses.. J Insect Physiol.

[51] Fares MA, Barrio E, Sabater-Munoz B, Moya A (2002). The evolution of the heat-shock protein GroEL from Buchnera, the primary endosymbiont of aphids, is governed by positive selection.. Mol Biol Evol.

[52] Fares MA, Ruiz-Gonzalez MX, Moya A, Elena SF, Barrio E (2002). Endosymbiotic bacteria: groEL buffers against deleterious mutations.. Nature.

[53] Dasch GA (1975). Morphological and molecular studies on intracellular bacterial symbiotes of insects.

[54] Holldobler B, Wilson EO (1990). The Ants.

[55] Bolton B (1994). Identification Guide to the Ant Genera of the World.

[56] Davidson DW (1997). The role of resource imbalances in the evolutionary ecology of tropical arboreal ants.. Biological Journal of the Linnean Society.

[57] Davidson DW (1998). Resource discovery versus resource domination in ants: a functional mechanism for breaking the trade-off.. Ecol Entomol.

[58] Gu X (1999). Statistical methods for testing functional divergence after gene duplication.. Mol Biol Evol.

[59] Gu X (2001). Maximum-likelihood approach for gene family evolution under functional divergence.. Mol Biol Evol.

[60] Gu X (2006). A simple statistical method for estimating type-II (cluster-specific) functional divergence of protein sequences.. Mol Biol Evol.

[61] Lopez P, Forterre P, Philippe H (1999). The root of the tree of life in the light of the covarion model.. J Mol Evol.

[62] Gao X, Vander Velden KA, Voytas DF, Gu X (2005). SplitTester: software to identify domains responsible for functional divergence in protein family.. BMC Bioinformatics.

[63] Gil R, Silva FJ, Zientz E, Delmotte F, Gonzalez-Candelas F (2003). The genome sequence of Blochmannia floridanus: comparative analysis of reduced genomes.. Proc Natl Acad Sci U S A.

[64] Feldhaar H, Straka J, Krischke M, Berthold K, Stoll S (2007). Nutritional upgrading for omnivorous carpenter ants by the endosymbiont Blochmannia.. BMC Biol.

[65] Goetz M, Bubert A, Wang G, Chico-Calero I, Vazquez-Boland JA (2001). Microinjection and growth of bacteria in the cytosol of mammalian host cells.. Proc Natl Acad Sci U S A.

[66] Thompson JD, Higgins DG, Gibson TJ (1994). CLUSTAL W: improving the sensitivity of progressive multiple sequence alignment through sequence weighting, position-specific gap penalties and weight matrix choice.. Nucleic Acids Res.

[67] Yang Z (2002). Inference of selection from multiple species alignments.. Curr Opin Genet Dev.

[68] Lynn DJ, Lloyd AT, Fares MA, O'Farrelly C (2004). Evidence of positively selected sites in mammalian alpha-defensins.. Mol Biol Evol.

[69] Chamary JV, Parmley JL, Hurst LD (2006). Hearing silence: non-neutral evolution at synonymous sites in mammals.. Nat Rev Genet.

[70] Parmley JL, Chamary JV, Hurst LD (2006). Evidence for purifying selection against synonymous mutations in mammalian exonic splicing enhancers.. Mol Biol Evol.

[71] Mayrose I, Doron-Faigenboim A, Bacharach E, Pupko T (2007). Towards realistic codon models: among site variability and dependency of synonymous and non-synonymous rates.. Bioinformatics.

[72] Resch AM, Carmel L, Marino-Ramirez L, Ogurtsov AY, Shabalina SA (2007). Widespread positive selection in synonymous sites of mammalian genes.. Mol Biol Evol.

[73] Yang Z (2007). PAML 4: phylogenetic analysis by maximum likelihood.. Mol Biol Evol.

[74] Nei M, Gojobori T (1986). Simple methods for estimating the numbers of synonymous and nonsynonymous nucleotide substitutions.. Mol Biol Evol.

[75] Ochman H, Wilson AC (1987). Evolution in bacteria: evidence for a universal substitution rate in cellular genomes.. J Mol Evol.

[76] Rispe C, Delmotte F, van Ham RC, Moya A (2004). Mutational and selective pressures on codon and amino acid usage in Buchnera, endosymbiotic bacteria of aphids.. Genome Res.

[77] Toft C, Fares MA (2009). Selection for translational robustness in Buchnera aphidicola, endosymbiotic bacteria of aphids.. Mol Biol Evol.

[78] Kanehisa M, Goto S (2000). KEGG: kyoto encyclopedia of genes and genomes.. Nucleic Acids Res.

[79] Tatusov RL, Koonin EV, Lipman DJ (1997). A genomic perspective on protein families.. Science.

