# Supplementary material for: Genome-Wide Functional Divergence after the Symbiosis of Proteobacteria with Insects Unraveled through a Novel Computational Approach
Source: PLoS Comput Biol. 2009 Apr 3;5(4):e1000344. doi: 10.1371/journal.pcbi.1000344 (PMC2659769; doi:10.1371/journal.pcbi.1000344)
Supplement: Table S1 — The ratio between the intensities of selection in the endosymbiont Buchnera aphidicola genomes and Blochmannia sp. and their free-living cousins. Genes are ordered alfabeticaly according to gene name in E. coli. Data missing or that could not be estimated are indicated by -. (0.70 MB DOC) [file pcbi.1000344.s001.doc]

**Table S1**: The ratio between the intensities of selection in the endosymbiont *Buchnera aphidicola* genomes and *Blochmannia sp*. and their free-living cousins. Genes are ordered alfabeticaly according to gene name in *E.coli*. Data missing or that could not be estimated are indicated by -.

|  | ***Buchnera aphidicola*** |  | ***Blochmannia*** |  |
| --- | --- | --- | --- | --- |
| **Gene Name** | **Gene tag** | **R(w)** | **Gene tag** | **R(w)** |
| **accA** | - | - | Bfl287 | 1.8625 |
| **accB** | - | - | Bfl292 | 1.7176 |
| **accC** | - | - | Bfl291 | 1.0699 |
| **accD** | - | - | Bfl495 | 5.0035 |
| **aceE** | BU205 | 0.2667 | Bfl153 | 0.4409 |
| **aceF** | BU206 | 1.3844 | Bfl152 | 2.5489 |
| **ackA** | BU175 | 4.0426 | - | - |
| **acpP** | - | - | Bfl403 | - |
| **acpS** | BU256 | 1.4595 | Bfl538 | 3.5473 |
| **adk** | BU484 | 5.8838 | Bfl302 | 2.4795 |
| **ahpC** | BU182 | 3.4344 | Bfl228 | 2.5909 |
| **alaS** | BU403 | 2.5206 | Bfl168 | 1.5093 |
| **amiB** | BU576 | 1.5 | Bfl078 | 2.0758 |
| **apaH** | BU142 | 2.6569 | Bfl125 | 3.5898 |
| **apt** | - | - | Bfl300 | 1.6016 |
| **argA** | BU456 | 0.4868 | - | - |
| **argB** | BU049 | 1.3579 | - | - |
| **argC** | BU048 | 1.0565 | - | - |
| **argD** | BU534 | 1.0398 | - | - |
| **argE** | BU047 | 1.1966 | - | - |
| **argG** | BU050 | 0.9732 | - | - |
| **argH** | BU051 | 2.4065 | - | - |
| **argI** | BU368 | 1.1507 | - | - |
| **argS** | BU242 | 1.1122 | Bfl453 | 3.2871 |
| **aroA** | BU311 | 4.7395 | Bfl382 | 1.0993 |
| **aroB** | BU538 | 0.3981 | Bfl571 | 1.1091 |
| **aroC** | BU097 | 1.703 | Bfl500 | 1.1748 |
| **aroE** | BU493 | 1.0417 | Bfl221 | 1.2674 |
| **aroF** | - | - | Bfl177 | 2.3809 |
| **aroH** | BU124 | 0.182 | - | - |
| **aroK** | BU539 | 1.4946 | Bfl572 | 1.2238 |
| **asd** | BU448 | 0.097 | Bfl574 | 2.2356 |
| **asnS** | BU360 | 13.1037 | Bfl421 | 1.4752 |
| **aspC** | - | - | Bfl422 | 4.4645 |
| **aspS** | BU316 | 1.007 | Bfl452 | 1.3331 |
| **atpA** | BU006 | 1.0085 | Bfl006 | 1.0318 |
| **atpB** | BU002 | 1.4646 | Bfl002 | 1.3305 |
| **atpC** | BU009 | 5.1775 | Bfl009 | 2.325 |
| **atpD** | BU008 | 0.4415 | Bfl008 | 2.0166 |
| **atpE** | BU003 | 0.026 | Bfl003 | - |
| **atpF** | BU004 | 6.7882 | Bfl004 | 1.9851 |
| **atpG** | BU007 | 0.8527 | Bfl007 | 1.5314 |
| **atpH** | BU005 | 3.9918 | Bfl005 | 1.7356 |
| **bacA** | BU062 | 2.324 | - | - |
| **bamA** | BU237 | 6.7606 | Bfl279 | 1.0752 |
| **bamD** | BU402 | 1.9108 | Bfl180 | 7.7873 |
| **bcp** | - | - | Bfl519 | 3.4791 |
| **bfr** | - | - | Bfl189 | 1.4112 |
| **bioA** | BU292 | 1.1824 | - | - |
| **bioB** | BU291 | 1.5955 | - | - |
| **birA** | - | - | Bfl184 | 6.5904 |
| **bolA** | BU473 | 4.189 | - | - |
| **carA** | BU145 | 0.4321 | Bfl122 | 1.1278 |
| **carB** | BU144 | 0.5502 | Bfl123 | 1.5929 |
| **cca** | BU061 | 2.0491 | Bfl062 | 1.1961 |
| **cdsA** | - | - | Bfl277 | 4.9211 |
| **clpB** | - | - | Bfl182 | 1.5722 |
| **clpP** | BU475 | 1.6416 | Bfl246 | 5.0082 |
| **clpX** | BU476 | 4.3777 | Bfl247 | 3.9695 |
| **cls** | BU273 | 1.1235 | Bfl433 | 3.0157 |
| **cmk** | - | - | Bfl381 | 3.3907 |
| **coaD** | BU583 | 0.3576 | - | - |
| **coaE** | BU203 | 2.3828 | - | - |
| **corA** | - | - | Bfl577 | 4.725 |
| **crr** | BU063 | 1.7664 | - | - |
| **cspC** | - | - | Bfl448 | - |
| **cspE** | BU489 | 0 | - | - |
| **csrA** | BU404 | - | Bfl169 | - |
| **cutA** | - | - | Bfl069 | 4.4978 |
| **cyaY** | BU590 | 0.9174 | - | - |
| **cyoA** | BU472 | 1.7422 | Bfl245 | 2.9116 |
| **cyoB** | BU471 | 1.2894 | Bfl244 | 1.7424 |
| **cyoC** | BU470 | 3.2871 | Bfl243 | 1.4638 |
| **cyoD** | BU469 | 1.4415 | Bfl242 | 5.5102 |
| **cyoE** | BU468 | 12.1649 | Bfl241 | 4.7241 |
| **cysA** | - | - | Bfl511 | 0.754 |
| **cysC** | BU422 | 0.8624 | Bfl164 | 0.7119 |
| **cysD** | - | - | Bfl162 | 2.4351 |
| **cysE** | BU054 | 0.8621 | Bfl603 | 3.7885 |
| **cysG** | - | - | Bfl161 | 1.0894 |
| **cysH** | - | - | Bfl160 | 3.225 |
| **cysI** | - | - | Bfl159 | 0.9842 |
| **cysJ** | BU428 | 2.4033 | Bfl158 | 0.9198 |
| **cysK** | BU066 | 1.0605 | Bfl508 | 1.4483 |
| **cysN** | - | - | Bfl163 | 0.9745 |
| **cysP** | - | - | Bfl514 | 2.1221 |
| **cysQ** | - | - | Bfl088 | 0.9904 |
| **cysS** | BU487 | 0.971 | Bfl304 | 1.8597 |
| **cysU** | - | - | Bfl513 | 2.0129 |
| **cysW** | - | - | Bfl512 | 5.9739 |
| **dapA** | BU096 | 1.2044 | Bfl518 | 0.4035 |
| **dapB** | BU146 | 1.3733 | Bfl121 | 1.9253 |
| **dapD** | BU229 | - | Bfl269 | 3.2312 |
| **dapE** | BU095 | 1.057 | Bfl517 | 3.6223 |
| **dapF** | BU589 | 1.8051 | Bfl579 | 2.0278 |
| **dcd** | BU108 | 6.2783 | - | - |
| **ddlA** | - | - | Bfl474 | 1.4776 |
| **deaD** | BU372 | 0.8346 | Bfl109 | 1.8551 |
| **def** | BU496 | 2.428 | Bfl219 | 5.1483 |
| **degP** | BU228 | 2.0795 | - | - |
| **degQ** | - | - | Bfl047 | 0.4886 |
| **deoB** | BU542 | 3.0813 | - | - |
| **deoD** | BU541 | 1.8112 | - | - |
| **der** | BU607 | 2.1125 | Bfl530 | 9.7607 |
| **dksA** | BU198 | 0.6316 | Bfl149 | 4.223 |
| **dnaA** | BU012 | 0.8115 | - | - |
| **dnaB** | BU546 | 0.4362 | Bfl027 | 0.8142 |
| **dnaC** | BU021 | 0.1999 | - | - |
| **dnaE** | BU238 | 3.324 | Bfl286 | 2.765 |
| **dnaG** | BU056 | 1.4321 | Bfl057 | 1.774 |
| **dnaJ** | BU152 | 0.6748 | Bfl115 | 1.0195 |
| **dnaK** | BU153 | 1.0098 | Bfl114 | 0.326 |
| **dnaN** | BU011 | 4.2396 | Bfl016 | 3.4324 |
| **dnaQ** | BU248 | 3.5979 | Bfl225 | 2.4277 |
| **dnaT** | BU022 | 6.9131 | - | - |
| **dnaX** | BU481 | 1.8541 | Bfl301 | 1.4296 |
| **dsbA** | BU430 | 1.814 | - | - |
| **dsbB** | - | - | Bfl438 | 4.4212 |
| **dut** | BU560 | 1.2498 | Bfl613 | 1.1508 |
| **dxr** | BU235 | 1.5237 | Bfl275 | 2.0037 |
| **dxs** | BU464 | 5.2331 | Bfl238 | 1.2431 |
| **efp** | BU020 | 0.8474 | Bfl072 | 16.7436 |
| **emrE** | - | - | Bfl550 | 0.2626 |
| **emtA** | - | - | Bfl391 | 1.9461 |
| **eno** | BU417 | 3.3537 | Bfl157 | 1.8501 |
| **era** | BU257 | 5.2604 | - | - |
| **erpA** | BU211 | 1.2074 | Bfl155 | 8.5612 |
| **fabA** | - | - | Bfl420 | 2.9375 |
| **fabB** | BU092 | 1.8163 | Bfl498 | 1.3316 |
| **fabD** | - | - | Bfl405 | 2.0179 |
| **fabG** | BU351 | 2.0836 | Bfl404 | 0.6201 |
| **fabH** | - | - | Bfl406 | 0.9383 |
| **fabI** | BU265 | 16.0127 | Bfl424 | 2.8026 |
| **fabZ** | - | - | Bfl282 | 2.3909 |
| **fbaA** | BU451 | 1.3925 | Bfl255 | 1.5952 |
| **fdx** | BU606 | 1.6842 | - | - |
| **ffh** | BU393 | 1.9448 | Bfl172 | 4.1697 |
| **fis** | BU400 | - | - | - |
| **fkpA** | BU533 | 3.7807 | - | - |
| **fldA** | BU299 | 0.6547 | Bfl325 | 2.1052 |
| **flgA** | BU336 | 2.8398 | - | - |
| **flgB** | BU337 | 1.4076 | - | - |
| **flgC** | BU338 | 1.0868 | - | - |
| **flgD** | BU339 | 1.4952 | - | - |
| **flgE** | BU340 | 2.8153 | - | - |
| **flgF** | BU341 | 2.4954 | - | - |
| **flgG** | BU342 | 0.2239 | - | - |
| **flgH** | BU343 | 0.5214 | - | - |
| **flgI** | BU344 | 1.9128 | - | - |
| **flgJ** | BU345 | 4.995 | - | - |
| **flgK** | BU346 | 1.9655 | - | - |
| **flhA** | BU241 | 0.6315 | - | - |
| **flhB** | BU240 | 1.0783 | - | - |
| **fliE** | BU072 | 0.5459 | - | - |
| **fliF** | BU073 | 2.2713 | - | - |
| **fliG** | BU074 | 1.2354 | - | - |
| **fliH** | BU075 | 3.994 | - | - |
| **fliI** | BU076 | 3.5484 | - | - |
| **fliK** | BU079 | 7.2897 | - | - |
| **fliM** | BU080 | 5.1252 | - | - |
| **fliN** | BU081 | 1.1505 | - | - |
| **fliP** | BU082 | 0.1685 | - | - |
| **fliQ** | BU083 | 0.5533 | - | - |
| **fliR** | BU084 | 0.7284 | - | - |
| **fmt** | BU497 | 1.8891 | Bfl218 | 2.7972 |
| **folA** | BU143 | 0.5407 | Bfl124 | 8.9582 |
| **folB** | - | - | Bfl061 | 2.8674 |
| **folC** | BU167 | 2.1924 | Bfl494 | 0.8351 |
| **folD** | BU486 | 1.4188 | Bfl305 | 1.4697 |
| **folE** | - | - | Bfl472 | 1.289 |
| **folK** | - | - | Bfl150 | 0.9671 |
| **folP** | - | - | Bfl099 | 1.7425 |
| **fpr** | BU581 | 4.9534 | Bfl600 | 3.3689 |
| **frr** | BU234 | 1.6787 | Bfl274 | 1.9004 |
| **ftsA** | BU213 | 1.6473 | Bfl145 | 6.3522 |
| **ftsB** | - | - | Bfl165 | 1.4438 |
| **ftsI** | BU222 | 1.7126 | Bfl136 | 1.5656 |
| **ftsK** | - | - | Bfl386 | 4.1955 |
| **ftsL** | BU223m | - | Bfl135 | 19.2767 |
| **ftsQ** | - | - | Bfl144 | 2.651 |
| **ftsW** | BU217 | 0.5305 | Bfl141 | 1.6416 |
| **ftsY** | BU024 | 3.4067 | Bfl627 | 6.6791 |
| **ftsZ** | BU212 | 0.8491 | Bfl146 | 1.7049 |
| **fumC** | - | - | Bfl373 | 1.6405 |
| **fusA** | BU527 | 0.5931 | Bfl565 | 0.2266 |
| **gapA** | BU298 | 1.6978 | Bfl437 | 1.6258 |
| **glmM** | - | - | Bfl100 | 1.3923 |
| **glmS** | BU026 | 1.4445 | - | - |
| **glmU** | BU027 | 0.9341 | Bfl010 | 1.5112 |
| **glnA** | - | - | Bfl618 | 3.5547 |
| **glnS** | BU415 | 1.3657 | Bfl324 | 1.6813 |
| **gloB** | BU246 | 3.0345 | Bfl223 | 1.3876 |
| **glpF** | BU306 | 3.5141 | - | - |
| **gltP** | - | - | Bfl030 | 0.7466 |
| **gltX** | BU070 | 1.5507 | Bfl504 | 2.1138 |
| **glyA** | BU289 | 3.2454 | Bfl536 | 0.4708 |
| **glyQ** | BU136 | 0.6578 | Bfl020 | 5.6059 |
| **glyS** | BU135 | 0.784 | Bfl021 | 1.4691 |
| **gmk** | BU434 | 0.8512 | Bfl616 | 1.4001 |
| **gnd** | BU107 | 2.1839 | Bfl470 | 2.974 |
| **gntY** | BU544 | 2.0424 | Bfl573 | 5.6098 |
| **gpmA** | BU304 | 2.7551 | Bfl342 | 4.9283 |
| **gpsA** | - | - | Bfl604 | 2.5578 |
| **gpt** | BU251 | 0.9366 | - | - |
| **greA** | BU384 | 0.6463 | Bfl096 | 3.0632 |
| **groL** | BU019 | 0.163 | Bfl071 | 0.1461 |
| **groS** | BU018 | 0.2127 | Bfl070 | 0.1808 |
| **grpE** | BU184 | 10.8196 | Bfl544 | 1.2855 |
| **grxC** | - | - | Bfl605 | 1.8098 |
| **grxD** | BU187 | 1.8484 | Bfl367 | 4.5832 |
| **gshA** | BU407 | 2.9517 | - | - |
| **gshB** | BU547 | 0.5691 | - | - |
| **guaA** | - | - | Bfl527 | 2.4614 |
| **guaB** | - | - | Bfl528 | 1.2593 |
| **guaC** | BU204 | 1.014 | - | - |
| **gutQ** | - | - | Bfl459 | 1.0383 |
| **gyrA** | BU180 | 0.3166 | Bfl476 | 0.3258 |
| **gyrB** | BU010 | 4.6543 | Bfl017 | 0.7504 |
| **hemC** | - | - | Bfl580 | 1.4391 |
| **hemD** | - | - | Bfl581 | 0.7361 |
| **hflB** | BU382 | 1.1125 | Bfl098 | 0.6277 |
| **hflC** | BU567 | 1.1995 | Bfl082 | 1.1316 |
| **hflK** | BU568 | 1.878 | Bfl081 | 1.1411 |
| **hflX** | - | - | Bfl080 | 2.2616 |
| **hinT** | BU357 | 1.5741 | Bfl398 | 9.5478 |
| **hisA** | BU104 | 1.631 | Bfl467 | 2.2212 |
| **hisB** | BU102 | 2.2452 | Bfl465 | 2.7882 |
| **hisC** | BU101 | 0.6125 | Bfl464 | 0.5942 |
| **hisD** | BU100 | 0.7141 | Bfl463 | 2.8603 |
| **hisF** | BU105 | 2.4562 | Bfl468 | 1.9181 |
| **hisG** | BU099 | 0.582 | Bfl462 | 0.7161 |
| **hisH** | BU103 | 2.8849 | Bfl466 | 4.3281 |
| **hisI** | BU106 | 1.1634 | Bfl469 | 1.3205 |
| **hisS** | BU288 | 0.889 | Bfl531 | 2.316 |
| **holA** | BU445 | 3.0801 | Bfl311 | 6.5685 |
| **holB** | BU354 | 0.8841 | Bfl400 | 1.0286 |
| **holC** | - | - | Bfl034 | 1.387 |
| **holD** | - | - | Bfl110 | 0.9834 |
| **hpt** | BU195 | 1.631 | - | - |
| **hscA** | BU605 | 1.439 | - | - |
| **hscB** | BU604 | 2.1689 | - | - |
| **hslU** | BU579 | 1.3351 | - | - |
| **hslV** | BU578 | 0.1991 | - | - |
| **hspQ** | - | - | Bfl419 | 0.4136 |
| **htpG** | BU483 | 0.66 | - | - |
| **htpX** | BU321 | 0.5354 | - | - |
| **hupA** | BU032 | 0.7462 | - | - |
| **ibpA** | BU580 | 0.3721 | Bfl018 | 1.3909 |
| **ihfA** | BU131 | 1.9212 | - | - |
| **ihfB** | BU308 | 6.1972 | - | - |
| **ileS** | BU149 | 2.5395 | Bfl118 | 0.437 |
| **ilvA** | - | - | Bfl589 | 0.9512 |
| **ilvC** | BU599 | 3.9774 | Bfl588 | 2.8094 |
| **ilvD** | BU600 | 1.1928 | Bfl590 | 0.9158 |
| **ilvE** | - | - | Bfl591 | 4.0895 |
| **ilvH** | BU225 | 0.8034 | - | - |
| **ilvI** | BU226 | 0.3524 | - | - |
| **ilvM** | - | - | Bfl592 | 1.7683 |
| **imp** | - | - | Bfl129 | 5.9363 |
| **infA** | BU315 | - | Bfl388 | - |
| **infB** | BU377 | 3.7773 | Bfl104 | 1.1373 |
| **infC** | BU126 | 1.5304 | Bfl352 | 2.7882 |
| **iscS** | BU602 | 0.8851 | Bfl534 | 2.6686 |
| **iscU** | BU603 | 0.3632 | - | - |
| **ispA** | BU465 | 2.0181 | - | - |
| **ispB** | - | - | Bfl092 | 4.4958 |
| **ispD** | BU420 | 2.2244 | - | - |
| **ispE** | BU170 | 2.3249 | Bfl347 | 1.919 |
| **ispF** | BU419 | 0.8853 | - | - |
| **ispG** | BU287 | 2.338 | - | - |
| **ispH** | BU147 | 6.0596 | - | - |
| **ispU** | BU236 | 1.8687 | Bfl276 | 0.4158 |
| **kdsA** | - | - | Bfl350 | 1.117 |
| **kdsB** | - | - | Bfl376 | 7.6396 |
| **ksgA** | BU141 | 4.4594 | Bfl126 | 2.8189 |
| **lepA** | BU260 | 5.8031 | Bfl542 | 1.5389 |
| **lepB** | BU259 | 4.7285 | Bfl541 | 3.8903 |
| **leuA** | - | - | Bfl133 | 1.0967 |
| **leuB** | - | - | Bfl132 | 1.8213 |
| **leuC** | - | - | Bfl131 | 1.3385 |
| **leuD** | - | - | Bfl130 | 2.9009 |
| **leuS** | BU444 | 1.0928 | Bfl313 | 2.3348 |
| **lgt** | - | - | Bfl265 | 4.0563 |
| **ligA** | - | - | Bfl507 | 2.9065 |
| **lipA** | BU269 | 1.4075 | - | - |
| **lipB** | BU268 | 1.7577 | - | - |
| **lnt** | - | - | Bfl314 | 3.3908 |
| **lolA** | - | - | Bfl385 | 5.8939 |
| **lolC** | BU295 | 4.8113 | Bfl396 | 3.7811 |
| **lolD** | BU296 | 27.451 | Bfl395 | 5.7015 |
| **lolE** | - | - | Bfl394 | 4.8359 |
| **lon** | BU477 | 0.5383 | Bfl299 | 3.814 |
| **lpcA** | BU250 | 1.3158 | Bfl226 | 3.6526 |
| **lpd** | BU207 | 3.1649 | Bfl151 | 3.4926 |
| **lplA** | - | - | Bfl357 | 2.5277 |
| **lpp** | - | - | Bfl364 | 0.3025 |
| **lptA** | - | - | Bfl043 | 5.506 |
| **lptB** | - | - | Bfl042 | 3.8505 |
| **lpxA** | - | - | Bfl283 | 0.2969 |
| **lpxB** | - | - | Bfl284 | 3.282 |
| **lpxC** | - | - | Bfl147 | 2.8589 |
| **lpxD** | - | - | Bfl281 | 2.036 |
| **lpxH** | - | - | Bfl303 | 1.0705 |
| **lpxK** | - | - | Bfl378 | 0.4284 |
| **lpxL** | - | - | Bfl411 | 1.3973 |
| **lspA** | BU148 | 7.5943 | Bfl119 | 1.9797 |
| **lysA** | BU438 | 1.8099 | Bfl263 | 0.7215 |
| **lysS** | - | - | Bfl262 | 1.4495 |
| **manX** | - | - | Bfl445 | 3.3058 |
| **manY** | - | - | Bfl446 | 1.2162 |
| **manZ** | - | - | Bfl447 | 0.5527 |
| **map** | BU230 | 0.5672 | Bfl270 | 0.4674 |
| **mdlA** | BU479 | 0.63 | - | - |
| **mdlB** | BU480 | 0.366 | - | - |
| **mdtH** | - | - | Bfl455 | 1.7916 |
| **metA** | - | - | Bfl630 | 4.9091 |
| **metB** | - | - | Bfl598 | 1.106 |
| **metC** | - | - | Bfl067 | 0.6399 |
| **metE** | BU030 | 1.8997 | Bfl625 | 2.2788 |
| **metF** | BU046 | 1.582 | Bfl597 | 1.8336 |
| **metG** | BU109 | 2.0295 | Bfl471 | 2.9968 |
| **metK** | BU408 | 0.6961 | Bfl252 | 2.0589 |
| **miaA** | - | - | Bfl079 | 1.747 |
| **miaB** | BU441 | 1.6616 | - | - |
| **minC** | BU327 | 2.0078 | Bfl439 | 0.4956 |
| **minD** | BU326 | 0.6849 | Bfl440 | 1.3296 |
| **minE** | BU325 | - | Bfl441 | 14.6248 |
| **mltA** | BU458 | 0.5567 | - | - |
| **mnmA** | BU261 | 0.7114 | Bfl392 | 2.7862 |
| **mnmE** | BU016 | 1.4767 | Bfl011 | 2.3592 |
| **mnmG** | BU001 | 0.9644 | Bfl001 | 3.0982 |
| **mnmG** | BU001 | 0.9644 | Bfl001 | 3.0982 |
| **mntH** | - | - | Bfl502 | 1.2957 |
| **mraW** | BU224 | 1.1 | Bfl134 | 2.8974 |
| **mraY** | - | - | Bfl139 | 9.227 |
| **mrcB** | - | - | Bfl154 | 1.9333 |
| **mrdA** | - | - | Bfl309 | 3.6239 |
| **mrdB** | - | - | Bfl308 | 6.8342 |
| **mreB** | - | - | Bfl294 | - |
| **mreC** | - | - | Bfl295 | 7.79 |
| **mreD** | - | - | Bfl296 | 4.2962 |
| **msbA** | - | - | Bfl379 | 3.0256 |
| **mscS** | BU452 | 0.8591 | - | - |
| **mtlA** | BU572 | 5.2281 | - | - |
| **mtlD** | BU571 | 2.1533 | - | - |
| **mtn** | BU210 | 3.1216 | - | - |
| **murA** | BU386 | 2.7587 | Bfl046 | 1.3999 |
| **murB** | BU045 | 1.4349 | Bfl183 | 0.6962 |
| **murC** | - | - | Bfl143 | 3.6442 |
| **murD** | BU218 | 0.4853 | Bfl140 | 1.9973 |
| **murE** | - | - | Bfl137 | 1.0703 |
| **murF** | - | - | Bfl138 | 0.8684 |
| **murG** | BU216 | 1.9645 | Bfl142 | 2.7884 |
| **murI** | BU554 | 2.2419 | - | - |
| **mutL** | BU570 | 3.4868 | - | - |
| **mutS** | BU429 | 0.8782 | - | - |
| **mutT** | BU202 | 1.054 | - | - |
| **mutY** | BU552 | 1.9967 | Bfl249 | 2.5478 |
| **nadD** | BU446 | 0.4776 | - | - |
| **nadE** | BU174 | 2.2559 | - | - |
| **nadK** | BU185 | 1.2034 | Bfl545 | 3.2891 |
| **nagA** | - | - | Bfl322 | 1.0272 |
| **nagB** | - | - | Bfl323 | 1.3398 |
| **ndk** | - | - | Bfl533 | 1.7966 |
| **nfo** | BU137 | 0.259 | - | - |
| **nlpD** | - | - | Bfl167 | 1.0938 |
| **nrdA** | BU179 | 0.7532 | Bfl478 | 0.9512 |
| **nrdB** | BU178 | 0.6237 | Bfl479 | 5.7614 |
| **nth** | BU119 | 0.5723 | Bfl372 | 4.0889 |
| **nuoA** | BU154 | 1.2292 | Bfl493 | 0.551 |
| **nuoB** | BU155 | 1.3867 | Bfl492 | 10.222 |
| **nuoC** | BU156 | 1.5673 | Bfl491 | 0.6446 |
| **nuoE** | BU157 | 2.0151 | Bfl490 | 4.9615 |
| **nuoF** | BU158 | 1.2594 | Bfl489 | 4.9366 |
| **nuoG** | BU159 | 9.5525 | Bfl488 | 1.1807 |
| **nuoH** | BU160 | 1.5851 | Bfl487 | 2.2605 |
| **nuoI** | BU161 | 0.9939 | Bfl486 | 9.0005 |
| **nuoJ** | BU162 | 0.3893 | Bfl485 | 4.4213 |
| **nuoK** | BU163 | 0.4826 | Bfl484 | 2.1487 |
| **nuoL** | BU164 | 3.5269 | Bfl483 | 3.3577 |
| **nuoM** | BU165 | 2.1023 | Bfl482 | 4.302 |
| **nuoN** | BU166 | 6.4155 | Bfl481 | 3.9284 |
| **nupC** | - | - | Bfl503 | 5.9146 |
| **nusA** | BU378 | 1.8045 | Bfl103 | 1.1053 |
| **nusB** | BU463 | 1.3217 | Bfl236 | 4.9236 |
| **nusG** | BU039 | 0.8502 | Bfl562 | 1.9666 |
| **obgE** | BU389 | 1.115 | Bfl095 | 3.2274 |
| **ompA** | BU332 | 0.8114 | - | - |
| **orn** | BU574 | 1.3711 | Bfl075 | 2.3088 |
| **pabA** | - | - | Bfl568 | 0.6035 |
| **pabB** | - | - | Bfl443 | 0.8549 |
| **pabC** | - | - | Bfl402 | 0.4882 |
| **pal** | - | - | Bfl339 | 3.9309 |
| **panB** | BU197 | 0.7898 | - | - |
| **panC** | BU196 | 0.9578 | - | - |
| **pdxA** | - | - | Bfl127 | 1.4859 |
| **pdxB** | - | - | Bfl497 | 1.1006 |
| **pdxH** | - | - | Bfl370 | 1.4692 |
| **pdxJ** | - | - | Bfl539 | 2.0459 |
| **pepA** | BU367 | 1.5143 | Bfl035 | 4.406 |
| **pfkA** | BU305 | 0.5822 | Bfl602 | 0.8235 |
| **pgi** | BU573 | 0.337 | Bfl629 | 1.4938 |
| **pgk** | BU450 | 3.0602 | Bfl254 | 0.9784 |
| **pgl** | BU293 | 1.3426 | Bfl341 | 2.1864 |
| **pgm** | - | - | Bfl326 | 4.3385 |
| **pgpA** | - | - | Bfl237 | 8.6958 |
| **pgsA** | - | - | Bfl415 | 6.9659 |
| **pheA** | BU392 | 0.3844 | Bfl179 | 3.381 |
| **pheS** | BU129 | 0.8741 | Bfl355 | 5.8299 |
| **pheT** | BU130 | 4.6255 | Bfl356 | 2.0966 |
| **pitA** | BU587 | 1.2185 | Bfl024 | 1.8053 |
| **plsC** | - | - | Bfl066 | 2.3973 |
| **plsX** | - | - | Bfl407 | 0.7333 |
| **pmbA** | BU089 | 1.5855 | Bfl298 | 2.7797 |
| **pncB** | BU361 | 1.0589 | - | - |
| **pnp** | BU373 | 0.0562 | Bfl108 | 0.7165 |
| **polA** | BU431 | 1.7726 | Bfl619 | 3.3132 |
| **ppa** | BU088 | 1.5814 | Bfl091 | 0.6045 |
| **ppiD** | BU478 | 3.4928 | - | - |
| **prfA** | BU171 | 0.4368 | Bfl348 | 3.9821 |
| **prfB** | - | - | Bfl261 | 1.6962 |
| **prfC** | BU543 | 1.3968 | - | - |
| **priA** | BU120 | 3.8178 | - | - |
| **prlC** | - | - | Bfl023 | 2.6417 |
| **prmB** | - | - | Bfl499 | 4.2308 |
| **prmC** | BU172 | 1.4133 | Bfl349 | 1.13 |
| **proS** | BU239 | 0.4144 | Bfl289 | 3.7776 |
| **prs** | BU169 | 0.2503 | Bfl346 | 2.9929 |
| **psd** | - | - | Bfl074 | 1.7851 |
| **pssA** | - | - | Bfl551 | 2.2457 |
| **pta** | BU176 | 2.7279 | - | - |
| **pth** | BU190 | 1.3227 | Bfl345 | 1.934 |
| **ptsG** | BU356 | 2.1578 | - | - |
| **ptsH** | BU065 | 0.024 | Bfl509 | - |
| **ptsI** | BU064 | 2.8186 | Bfl510 | 1.0802 |
| **purA** | BU566 | 3.2496 | Bfl083 | 1.6593 |
| **purB** | BU263 | 0.8911 | Bfl393 | 2.2236 |
| **purH** | BU031 | 0.355 | Bfl555 | 1.8038 |
| **pykA** | BU319 | 1.8406 | Bfl450 | 4.7988 |
| **pyrB** | BU369 | 0.4504 | - | - |
| **pyrC** | BU334 | 1.5994 | - | - |
| **pyrD** | BU362 | 1.3359 | - | - |
| **pyrF** | BU270 | 0.4151 | - | - |
| **pyrG** | BU416 | 0.363 | Bfl156 | 0.703 |
| **pyrH** | BU233 | 4.9206 | Bfl273 | 2.4605 |
| **pyrI** | BU370 | 1.8592 | - | - |
| **queA** | BU132 | 0.9081 | - | - |
| **rbfA** | BU376 | 12.0486 | Bfl105 | 3.0988 |
| **recB** | BU454 | 6.275 | Bfl268 | 2.7051 |
| **recC** | BU453 | 0.8833 | Bfl266 | 3.9784 |
| **recD** | BU455 | 1.4643 | Bfl267 | 1.9675 |
| **rep** | BU598 | 0.4947 | - | - |
| **rfaC** | - | - | Bfl609 | 0.974 |
| **rfaD** | - | - | Bfl607 | 2.361 |
| **rfaE** | BU060 | 0.5215 | Bfl063 | 3.5312 |
| **rfaF** | - | - | Bfl608 | 3.6493 |
| **rho** | BU596 | 0.0868 | Bfl586 | 1.058 |
| **ribA** | BU271 | 0.7023 | Bfl425 | 1.3746 |
| **ribB** | BU059 | 1.182 | Bfl065 | 2.1123 |
| **ribC** | BU112 | 0.6747 | Bfl366 | 4.2622 |
| **ribD** | BU462 | 1.2878 | Bfl234 | 1.6448 |
| **ribE** | BU459 | 1.3517 | Bfl235 | 0.6228 |
| **ribF** | BU150 | 0.941 | Bfl117 | 1.0721 |
| **rimM** | BU395 | 4.9689 | Bfl174 | 1.3936 |
| **rimN** | BU494 | 2.8011 | Bfl220 | 0.6391 |
| **rlmB** | - | - | Bfl084 | 7.099 |
| **rlmL** | BU363 | 0.7947 | - | - |
| **rlmN** | BU286 | 5.4555 | - | - |
| **rlpB** | - | - | Bfl312 | 3.6674 |
| **rluB** | BU282 | 3.3176 | - | - |
| **rluC** | BU348 | 2.8134 | Bfl409 | 6.0811 |
| **rluD** | BU401 | 2.2125 | Bfl181 | 2.7153 |
| **rmuC** | - | - | Bfl623 | 1.6107 |
| **rnb** | BU266 | 0.7387 | - | - |
| **rnc** | BU258 | 0.6287 | Bfl540 | 2.5407 |
| **rne** | BU347 | 2.8097 | Bfl410 | 0.7982 |
| **rnhA** | - | - | Bfl224 | 1.0664 |
| **rnhB** | - | - | Bfl285 | 3.8863 |
| **rnpA** | BU014 | 6.0887 | Bfl014 | 1.3587 |
| **rnr** | BU565 | 1.3064 | - | - |
| **rnt** | BU188 | 0.5344 | Bfl368 | 0.9732 |
| **rpe** | BU537 | 1.7173 | Bfl570 | 3.7287 |
| **rpiA** | BU411 | 1.2656 | Bfl256 | 3.5189 |
| **rplA** | BU037 | 1.0484 | Bfl560 | 1.4217 |
| **rplB** | BU521 | - | Bfl194 | 0.9185 |
| **rplC** | BU524 | 3.2874 | Bfl191 | 1.2503 |
| **rplD** | BU523 | - | Bfl192 | - |
| **rplE** | BU512 | - | Bfl203 | 0.4175 |
| **rplF** | BU509 | 0.738 | Bfl206 | 2.0563 |
| **rplI** | BU562 | 0.7261 | Bfl087 | 0.6667 |
| **rplJ** | BU036 | 8.7901 | Bfl559 | 0.6441 |
| **rplK** | BU038 | 0.0193 | Bfl561 | - |
| **rplL** | BU035 | 0.489 | Bfl558 | 0.5981 |
| **rplM** | BU391 | - | Bfl049 | - |
| **rplN** | BU514 | - | Bfl201 | 0.2909 |
| **rplO** | BU505 | - | Bfl210 | 0.9844 |
| **rplP** | BU517 | - | Bfl198 | 0.2843 |
| **rplQ** | BU498 | 3.9902 | Bfl217 | 1.2132 |
| **rplR** | BU508 | - | Bfl207 | 0.4679 |
| **rplS** | BU397 | - | Bfl176 | 0.2454 |
| **rplT** | BU128 | - | Bfl354 | - |
| **rplU** | BU387 | - | Bfl093 | 1.5833 |
| **rplV** | BU519 | - | Bfl196 | - |
| **rplW** | BU522 | 7.0554 | Bfl193 | 1.7477 |
| **rplX** | BU513 | - | Bfl202 | - |
| **rplY** | BU138 | 4.6258 | Bfl473 | 1.2948 |
| **rpmA** | BU388 | 1.1734 | Bfl094 | 0.7999 |
| **rpmB** | BU086 | 48.2451 | Bfl612 | 0.1924 |
| **rpmC** | BU516 | 0.438 | - | - |
| **rpmD** | BU506 | - | - | - |
| **rpmE** | BU577 | 1.4724 | Bfl599 | 0.3835 |
| **rpmF** | BU349 | - | Bfl408 | - |
| **rpmG** | BU085 | 0.8315 | Bfl611 | - |
| **rpmH** | BU013 | 0.0345 | Bfl015 | - |
| **rpmI** | BU127 | - | Bfl353 | - |
| **rpmJ** | - | - | Bfl212 | - |
| **rpoA** | BU499 | - | Bfl216 | 1.9913 |
| **rpoB** | BU034 | 1.5232 | Bfl557 | 0.6027 |
| **rpoC** | BU033 | 2.3439 | Bfl556 | 0.3617 |
| **rpoD** | BU055 | 0.3418 | Bfl056 | 1.3508 |
| **rpoH** | BU025 | 0.6248 | Bfl626 | 1.7321 |
| **rpoZ** | - | - | Bfl617 | - |
| **rppH** | - | - | Bfl264 | 5.7691 |
| **rpsA** | BU309 | - | Bfl380 | 1.6261 |
| **rpsB** | BU231 | 1.3246 | Bfl271 | 0.453 |
| **rpsC** | BU518 | - | Bfl197 | - |
| **rpsD** | BU500 | - | Bfl215 | 0.8648 |
| **rpsE** | BU507 | - | Bfl208 | - |
| **rpsF** | BU564 | 2.669 | Bfl085 | 5.0405 |
| **rpsG** | BU528 | 0.8941 | Bfl566 | 1.5068 |
| **rpsH** | BU510 | - | Bfl205 | - |
| **rpsI** | BU390 | - | Bfl050 | 0.6119 |
| **rpsJ** | BU525 | 0.9286 | Bfl190 | 0.2526 |
| **rpsK** | BU501 | - | Bfl214 | 0.8211 |
| **rpsL** | BU529 | - | Bfl567 | - |
| **rpsM** | BU502 | 0.2 | Bfl213 | 0.3123 |
| **rpsN** | BU511 | 0.8544 | Bfl204 | 0.6411 |
| **rpsO** | BU374 | 1.5536 | Bfl107 | 1.2504 |
| **rpsP** | BU394 | 18.0461 | Bfl173 | 1.6644 |
| **rpsQ** | BU515 | - | Bfl200 | 1.6058 |
| **rpsR** | BU563 | 0.3447 | Bfl086 | 0.0711 |
| **rpsS** | BU520 | - | Bfl195 | 0.1229 |
| **rpsT** | BU151 | - | Bfl116 | 0.1397 |
| **rpsU** | BU057 | 0.0273 | Bfl058 | - |
| **rrmJ** | BU383 | - | Bfl097 | - |
| **rseP** | - | - | Bfl278 | 1.178 |
| **rsmC** | BU328 | 1.6599 | - | - |
| **rsmD** | - | - | Bfl628 | 0.8071 |
| **rsmE** | BU410 | 2.6106 | - | - |
| **rsxA** | BU113 | 1.0418 | - | - |
| **rsxB** | BU114 | 2.0371 | - | - |
| **rsxD** | BU116 | 1.9482 | - | - |
| **rsxE** | BU118 | 0.9453 | - | - |
| **rsxG** | BU117 | 1.5507 | - | - |
| **sbcB** | BU555 | 1.0899 | Bfl461 | 4.1395 |
| **sdhA** | - | - | Bfl329 | - |
| **sdhB** | - | - | Bfl330 | 2.7883 |
| **sdhC** | - | - | Bfl327 | 2.778 |
| **sdhD** | - | - | Bfl328 | 5.9253 |
| **secA** | BU201 | 1.0018 | Bfl148 | 1.0368 |
| **secB** | BU053 | 4.9946 | - | - |
| **secD** | - | - | Bfl232 | 1.5942 |
| **secE** | BU040 | 2.438 | Bfl563 | 0.8959 |
| **secF** | - | - | Bfl233 | 1.7905 |
| **secY** | BU504 | 4.3841 | Bfl211 | 1.3349 |
| **serC** | BU312 | 0.2792 | Bfl383 | 1.885 |
| **serS** | BU313 | 1.3238 | Bfl384 | 2.838 |
| **sirA** | BU447 | 1.2348 | - | - |
| **skp** | - | - | Bfl280 | 0.7405 |
| **slyA** | - | - | Bfl369 | 0.977 |
| **smg** | BU495 | 1.4093 | - | - |
| **smpB** | BU254 | 3.9953 | Bfl548 | 5.1079 |
| **sodA** | BU189 | 1.6067 | Bfl022 | 3.3587 |
| **sohB** | BU283 | 1.5467 | - | - |
| **speB** | - | - | Bfl253 | 4.1955 |
| **speD** | BU208 | 0.3401 | - | - |
| **speE** | BU209 | 0.6563 | - | - |
| **sppA** | - | - | Bfl436 | 1.4857 |
| **ssb** | BU545 | 0.6502 | Bfl028 | 0.7515 |
| **sucA** | BU302 | 2.6649 | Bfl331 | 1.2291 |
| **sucB** | BU303 | 3.453 | Bfl332 | 2.6528 |
| **sucC** | - | - | Bfl333 | 7.8753 |
| **sucD** | - | - | Bfl334 | 0.853 |
| **sufA** | BU122 | 1.446 | Bfl358 | 2.2622 |
| **sufB** | - | - | Bfl359 | 1.4616 |
| **sufC** | - | - | Bfl360 | 1.4503 |
| **sufD** | - | - | Bfl361 | 1.0103 |
| **sufE** | - | - | Bfl363 | 2.2005 |
| **sufS** | - | - | Bfl362 | 1.7789 |
| **suhB** | BU285 | 2.0948 | Bfl535 | 7.9595 |
| **surA** | BU140 | 2.3388 | Bfl128 | 5.5702 |
| **tadA** | BU255 | 1.4596 | Bfl537 | 2.3668 |
| **talA** | BU093 | 1.321 | Bfl515 | 14.8186 |
| **tdk** | - | - | Bfl434 | 4.3449 |
| **tgt** | BU133 | 1.651 | Bfl230 | 2.3977 |
| **thiI** | - | - | Bfl239 | 3.4559 |
| **thiL** | BU460 | 0.7814 | - | - |
| **thrA** | BU194 | 8.2684 | Bfl111 | 1.4369 |
| **thrB** | BU193 | 0.4299 | Bfl112 | 1.6915 |
| **thrC** | BU192 | 1.5141 | Bfl113 | 1.4205 |
| **thrS** | BU125 | 3.3109 | Bfl351 | 1.9573 |
| **thyA** | BU440 | 1.6328 | - | - |
| **tig** | BU474 | 17.1857 | - | - |
| **tilS** | BU110 | 1.1326 | Bfl288 | 0.4451 |
| **tktA** | - | - | Bfl516 | 0.9037 |
| **tldD** | BU398 | 6.6276 | Bfl297 | 1.4751 |
| **tmk** | BU353 | 0.5166 | Bfl401 | 3.2605 |
| **tolA** | - | - | Bfl337 | 1.0386 |
| **tolB** | - | - | Bfl338 | 1.3649 |
| **tolQ** | - | - | Bfl335 | 9.8659 |
| **tolR** | - | - | Bfl336 | 2.2717 |
| **tonB** | - | - | Bfl432 | 2.6586 |
| **topA** | BU284 | 2.9928 | - | - |
| **tpiA** | BU307 | 1.8326 | Bfl601 | 1.7582 |
| **trmD** | BU396 | 5.6487 | Bfl175 | 0.762 |
| **trmI** | BU551 | 2.1276 | - | - |
| **trpA** | BU277 | 1.2686 | Bfl431 | 1.818 |
| **trpB** | BU278 | 0.9305 | Bfl430 | 2.0436 |
| **trpC** | BU279 | 4.283 | Bfl429 | 1.8954 |
| **trpD** | BU280 | 1.2587 | Bfl428 | 7.6268 |
| **trpE** | - | - | Bfl426 | 0.8543 |
| **trpS** | BU536 | 3.4352 | Bfl569 | 2.5766 |
| **truA** | BU199 | 1.5131 | Bfl496 | 4.4966 |
| **truB** | BU375 | 3.3839 | Bfl106 | 2.9753 |
| **trxA** | BU597 | 0.0059 | Bfl587 | - |
| **trxB** | BU314 | 4.5502 | Bfl387 | 3.9296 |
| **tsf** | BU232 | 2.0596 | Bfl272 | 1.7665 |
| **tsgA** | BU535 | 1.4742 | - | - |
| **typA** | BU433 | 2.0056 | - | - |
| **tyrA** | - | - | Bfl178 | 3.8158 |
| **tyrS** | BU121 | 0.487 | Bfl371 | 3.2791 |
| **ubiA** | - | - | Bfl025 | 2.9446 |
| **ubiB** | - | - | Bfl621 | 2.0854 |
| **ubiD** | - | - | Bfl620 | 1.4111 |
| **ubiE** | - | - | Bfl622 | 3.4757 |
| **ubiF** | - | - | Bfl318 | 3.0163 |
| **ubiG** | - | - | Bfl477 | 4.8768 |
| **ubiH** | - | - | Bfl259 | 1.087 |
| **ubiX** | - | - | Bfl375 | 1.4193 |
| **udp** | - | - | Bfl624 | 2.6381 |
| **ung** | - | - | Bfl543 | 3.4151 |
| **upp** | - | - | Bfl520 | 2.8476 |
| **uup** | BU364 | 1.1617 | - | - |
| **valS** | BU366 | 3.5253 | Bfl033 | 4.0808 |
| **waaA** | - | - | Bfl610 | 3.9215 |
| **xthA** | - | - | Bfl435 | 1.4429 |
| **yabI** | BU139 | 1.5333 | - | - |
| **yajC** | BU134 | 0.3871 | Bfl231 | 4.6249 |
| **yajR** | BU466 | 2.796 | Bfl240 | 3.3012 |
| **ybaB** | BU482 | - | - | - |
| **ybeB** | - | - | Bfl310 | 3.2078 |
| **ybeD** | BU488 | 2.2353 | - | - |
| **ybeX** | BU443 | 4.3857 | Bfl315 | 2.2309 |
| **ybeY** | BU442 | 0.4517 | Bfl316 | 2.2464 |
| **ybeZ** | - | - | Bfl317 | 13.0972 |
| **ybgF** | - | - | Bfl340 | 1.6221 |
| **ybgI** | BU301 | 1.6787 | - | - |
| **ybhL** | - | - | Bfl343 | 1.0881 |
| **ycaR** | - | - | Bfl377 | 3.879 |
| **ycbL** | - | - | Bfl423 | 1.4585 |
| **yccK** | BU467 | 2.6774 | Bfl418 | 2.091 |
| **yceA** | BU365 | 1.0565 | - | - |
| **yceN** | BU333 | 3.5679 | Bfl454 | 4.0276 |
| **ycfH** | BU355 | 1.0671 | Bfl399 | 2.3133 |
| **ycfM** | - | - | Bfl397 | 4.5378 |
| **ychA** | BU173 | 1.3373 | - | - |
| **ychE** | BU267 | 0.5516 | - | - |
| **ychF** | BU191 | 1.4525 | Bfl344 | 2.103 |
| **yciA** | BU274 | 1.5768 | - | - |
| **yciB** | BU275 | 0.7587 | - | - |
| **yciC** | BU276 | 1.0719 | - | - |
| **ydiK** | BU123 | 0.5669 | - | - |
| **yeaZ** | BU324 | 2.1507 | Bfl442 | 1.0261 |
| **yebA** | - | - | Bfl451 | 3.9694 |
| **yeeX** | BU556 | 0.405 | Bfl460 | 1.2738 |
| **yfaE** | - | - | Bfl480 | 7.7717 |
| **yfcN** | BU098 | 0.0961 | - | - |
| **yfgM** | BU608 | 1.7455 | - | - |
| **yfjF** | BU253 | 3.5847 | - | - |
| **yfjG** | - | - | Bfl547 | 8.9379 |
| **ygfA** | - | - | Bfl257 | 1.1724 |
| **ygfZ** | BU435 | 2.0778 | Bfl260 | 2.0147 |
| **yggS** | BU549 | 2.2723 | - | - |
| **yggW** | BU550 | 2.9773 | - | - |
| **yggX** | BU553 | 0.6813 | Bfl248 | 5.5153 |
| **ygiH** | - | - | Bfl060 | 3.4676 |
| **ygjD** | BU058 | 1.0533 | Bfl059 | 2.0003 |
| **yhcB** | - | - | Bfl048 | 16.4386 |
| **yheL** | BU530 | 2.9894 | - | - |
| **yheM** | BU531 | 3.7267 | - | - |
| **yheN** | BU532 | 0.825 | - | - |
| **yhiQ** | BU586 | 3.198 | - | - |
| **yibN** | BU052 | 3.3171 | Bfl606 | 6.7885 |
| **yicC** | - | - | Bfl614 | 4.9134 |
| **yidC** | BU015 | 4.0893 | Bfl012 | 3.5519 |
| **yidZ** | - | - | Bfl038 | 1.8996 |
| **yigB** | - | - | Bfl578 | 1.2588 |
| **yigL** | BU028 | 1.289 | Bfl576 | 1.1679 |
| **yihA** | BU432 | 1.9185 | - | - |
| **yjcE** | - | - | Bfl029 | 1.4102 |
| **yjeE** | - | - | Bfl077 | 2.5647 |
| **yjeP** | - | - | Bfl073 | 3.7207 |
| **yjgF** | - | - | Bfl031 | 1.2081 |
| **yjgP** | - | - | Bfl036 | 2.7942 |
| **yjgQ** | - | - | Bfl037 | 10.2813 |
| **ynfM** | BU588 | 1.3501 | - | - |
| **yoaE** | BU323 | 1.2146 | Bfl444 | 1.3094 |
| **yqeI** | - | - | Bfl390 | 0.3913 |
| **yqgE** | - | - | Bfl251 | 1.0567 |
| **yqgF** | BU548 | 3.085 | Bfl250 | 8.928 |
| **yqjA** | - | - | Bfl054 | 6.2785 |
| **yraL** | BU091 | 0.2181 | Bfl052 | 1.5766 |
| **yraP** | - | - | Bfl051 | 2.6797 |
| **yrbA** | BU385 | 2.1609 | Bfl045 | 11.1041 |
| **yrbK** | - | - | Bfl044 | 3.6642 |
| **ytfF** | - | - | Bfl307 | 1.3315 |
| **ytfN** | BU087 | 1.6083 | Bfl090 | 4.3045 |
| **zapA** | - | - | Bfl258 | 2.2147 |
| **znuB** | BU317 | 0.486 | Bfl041 | 7.7383 |
| **znuC** | BU318 | 3.2159 | - | - |
| **zur** | - | - | Bfl026 | 4.194 |
| **zwf** | BU320 | 2.2083 | Bfl449 | 3.9884 |
