# Supplementary material for: Genome-Wide Functional Divergence after the Symbiosis of Proteobacteria with Insects Unraveled through a Novel Computational Approach
Source: PLoS Comput Biol. 2009 Apr 3;5(4):e1000344. doi: 10.1371/journal.pcbi.1000344 (PMC2659769; doi:10.1371/journal.pcbi.1000344)
Supplement: Table S2 — Functional divergence analysis in the metabolic pathways of Buchnera and Blochmannia endosymbionts. (0.09 MB DOC) [file pcbi.1000344.s002.doc]

**Table S2.** Functional divergence analysis in the metabolic pathways of *Buchnera* and *Blochmannia* endosymbionts.

| **Metab.** | **#Genes in *E.coli*** | **#FD *Buchnera*** | **#FD *Blochmannia*** |
| --- | --- | --- | --- |
| ABC transporters | 189 | 1** | 8** |
| Ala & Asp metab. | 28 | 4 | 8 |
| Aa-tRNA biosyn. | 25 | 16** | 23** |
| Aminosugars metab. | 18 | 0 | 6 |
| Arg & Pro metab. | 18 | 3 | 3 |
| Bacterial chemotaxis | 17 | 0 | - |
| Base excision repair | 14 | 1 | 6 |
| beta-Ala metab. | 14 | 0 | - |
| Biosyn. of steroids | 9 | 0 | 3 |
| Biosyn. of unsaturated FA | 4 | 2 | 1 |
| Biotin metab. | 7 | 0 | 1 |
| Butanoate metab. | 34 | 3 | 5* |
| C5-Branched dibasic acid metab. | 7 | - | 2 |
| Carbon fixation | 20 | 4 | 6 |
| Citrate cycle (TCA cycle) | 27 | 1* | 8* |
| Cys metab. | 13 | 1 | 6 |
| D-Gln & D-Glu metab. | 5 | 0 | 2 |
| DNA replication | 17 | 5* | 14** |
| Drug metab. - other enzymes | 10 | 0 | 4 |
| Fatty acid biosyn. | 12 | 3* | 8** |
| Flagellar assembly | 38 | 4 | 9 |
| Folate biosyn. | 14 | 0 | - |
| Fructose & mannose metab. | 47 | 2 | 6* |
| Galactose metab. | 32 | - | 2* |
| Glutamate metab. | 32 | 4 | 7 |
| Glutathione metab. | 14 | 1 | 3 |
| Glycan structures | 11 | - | - |
| Glycerophospholipid metab. | 27 | - | 8* |
| Gly, Ser & Thr metab. | 34 | 9* | 13* |
| Glycolysis / Gluconeogenesis | 37 | 6 | 10* |
| Glyoxylate & dicarboxylate metab. | 34 | 1* | 1* |
| His metab. | 12 | 3 | 9 |
| Homologous recombination | 27 | 6* | 12 |
| Inositol phosphate metab. | 4 | 1 | 1 |
| Lipoic acid metab. | 3 | 1 | 0 |
| Lipopolysaccharide biosyn. | 28 | 1 | 14** |
| Lys biosyn. | 18 | 8* | 10** |
| Lys degradation | 10 | 1 | 2 |
| Methane metab. | 13 | 2 | 2* |
| Met metab. | 16 | 2 | 7 |
| Mismatch repair | 22 | 4 | 10 |
| Nitrogen metab. | 35 | - | 2* |
| Novobiocin biosyn. | 3 | - | 1 |
| Nicotinate & nicotinamide metab. | 12 | 0 | 2 |
| Nucleotide excision repair | 8 | 0 | - |
| One carbon pool by folate | 12 | 4* | 6* |
| Oxidative phosphorylation | 41 | 13* | 25** |
| Pantothenate & CoA biosyn. | 17 | 4 | 4 |
| Pentose phosphate pathway | 29 | 5 | 10 |
| Peptidoglycan biosyn. | 17 | 0 | 10** |
| Phe metab. | 17 | - | 1 |
| Phe, Tyr & Trp biosyn. | 24 | 6* | 17** |
| Phosphotransferase system | 52 | 0** | 3** |
| Porphyrin & chlorophyll metab. | 23 | 3 | 5 |
| Propanoate metab. | 30 | 1 | 4 |
| Protein export | 18 | 4 | 7 |
| Purine metab. | 79 | 5 | 24 |
| Pyrimidine metab. | 48 | 6 | 25** |
| Pyruvate metab. | 42 | 3 | 9 |
| Reductive carboxylate cycle | 22 | - | 5 |
| Riboflavin metab. | 11 | 0 | 5 |
| Ribosome | 79 | 13* | 22 |
| RNA polymerase | 4 | 2* | 2* |
| Selenoamino acid metab. | 15 | 0 | 9* |
| Starch & sucrose metab. | 33 | - | 2** |
| Streptomycin biosyn. | 9 | - | 2 |
| Sulfur metab. | 13 | 0 | 10** |
| Taurine & hypotaurine metab. | 6 | 1 | 1 |
| Terpenoid biosyn. | 3 | 0 | - |
| Thiamine metab. | 15 | 1 | 2* |
| Trp metab. | 12 | 2 | 3 |
| Two-component system | 129 | 0** | 6** |
| Tyr metab. | 11 | 3 | 2 |
| Ubiquinone biosyn. | 30 | 0 | 20 |
| Urea cycle & metab. of amino groups | 28 | 2 | 0** |
| Val, Leu & Ile biosyn. | 19 | 7* | 13** |
| Val, Leu & Ile degradation | 11 | 0 | 2 |
| Vitamin B6 metab. | 9 | 2 | 6* |

Where biosyn. stands for biosynthesis, metab. stands for metabolism, FA stands for fatty acids, and amino acids are in three letter format. We indicated metabolic pathways in *Buchnera* or *Blochmannia* enriched or impoverished by functional divergent genes by asterisks (*, P < 0.05; **, P < 10^-3^).
